# Unpacking the Specific Associations Between Adverse Childhood Experiences and Depressive Symptoms among the Middle-Aged and Elderly Chinese Populations: A Dimensional Approach and Latent Class Analysis in a Cohort Study

**DOI:** 10.1155/2023/8439527

**Published:** 2023-08-02

**Authors:** Mengna Wei, Miyuan Wang, Rui Chang, Chunan Li, Ke Xu, Yanfen Jiang, Yimin Wang, Paiziyeti Tuerxun, Jianduan Zhang

**Affiliations:** ^1^Department of Maternal and Child Health, School of Public Health, Tongji Medical College, Huazhong University of Science and Technology, Wuhan, China; ^2^Key Laboratory of Environment and Health, Ministry of Education and Ministry of Environmental Protection, State Key Laboratory of Environmental Health (Incubating), School of Public Health, Tongji Medical College, Huazhong University of Science and Technology, 13 Hangkong Road, Wuhan, Hubei, 430030, China

## Abstract

**Background:**

Adverse childhood experiences (ACEs) are recognized as key risk factors linked to poor mental health throughout life. However, research on the specific associations between ACE dimensions and depressive symptoms (DSs) among the Chinese population during mid to late life is rare.

**Objectives:**

This study aims to investigate the specific effects of different dimensions of ACEs on the new occurrence of DSs and the number of times with DSs among the middle-aged and elderly Chinese populations.

**Methods:**

The analysis included 3979 adults aged ≥45 years with four repeated measurements of the 10-item Center for Epidemiological Studies Depression Scale from the China Health and Retirement Longitudinal Study 2011–2018. Three types of ACE dimensions (total, deprivation, and threat-related ACEs) were conceptualized in accordance with the 15 types of ACEs that occurred before the age of 18 years recorded using the Life History Survey Questionnaire in 2014. In addition, latent class analysis (LCA) was utilized as an additional method for identifying distinct ACE clusters. The Cox regression and ordered logistic regression were used to estimate the risk of ACEs on DSs.

**Results:**

Among the 3979 participants, 1656 developed a new occurrence of DSs during follow-up, with 998, 438, and 220 exhibiting DSs one, two, and three times. For total and deprivation-related ACEs, only the group with ≥3 ACEs was significant with the new occurrence of DSs when compared with the no ACE group, and the adjusted hazard ratios (HRs) (95% confidence interval) were 1.562 (1.296, 1.882) and 1.446 (1.221, 1.712), respectively. With regard to threat-related ACEs, all three groups (1 ACE, 2 ACEs, and ≥3 ACEs) were significantly associated with the new occurrence of DSs. The HRs were 1.260 (1.115, 1.425), 1.407 (1.212, 1.634), and 1.585 (1.366, 1.840), respectively. The findings of total, deprivation-related, and threat-related ACEs and their associations with the number of times with DSs represent a similar phenomenon. The LCA revealed five ACE clusters. Compared to the “low risk” cluster, the “poor parent relationship” cluster and the “physical abuse” cluster were linked to an increased risk of the new occurrence of DSs and the number of times with DSs. The results of the subgroup analysis by sex and age were consistent with the total population.

**Conclusions:**

Individuals who have experienced higher ACE scores in early life face a higher risk of developing a new occurrence of DSs and multiple detected DSs in mid to late life, particularly in the case of threat-related ACEs. Parsing ACEs is imperative to explore their distinct effects on DSs and the underlying mechanisms. In addition, Incorporating ACE screening into regular health checks among the middle-aged and elderly populations is recommended. Moreover, targeted mental health interventions should be delivered to those who have experienced early life adversities, particularly threat-related ACEs, to promote healthy aging.

## 1. Introduction

Depression, a prevalent but frequently undetected psychiatric disorder, is significantly associated with a diminished quality of life [1] and an increased economic burden due to treatment costs and related lost work productivity [2]. It has been ranked as the third disease burden worldwide and is expected to be on top by 2030 across all ages on the World Health Organization's list of medical conditions [3, 4]. Remarkably, the high prevalence of depression among those aged ≥45 years is becoming a growing public health concern. The baseline data of the China Health and Retirement Longitudinal Study (CHARLS) indicated that 30% of men and 45% of women aged 45 years and above had depressive symptoms (DSs). With the declining birth rate and increasing human life expectancy, China is facing an unprecedented population aging issue. In accordance with the 2019 China Statistical Yearbook, 17.9% of the total population is aged 60 years or older, representing 249.49 million people [5, 6]. The projected number for 2025 would reach 300 million, and about 400 million by 2050 [6, 7]. The rapid population aging in China poses severe challenges to the prevention and control of DSs. Moreover, multiple DSs are common in major depression. Results from nonclinical cohorts indicated that one-third of patients who experienced one episode would have another [8]. In clinical settings, over three-quarters of patients will have multiple DSs [9]. In addition, multiple depressive episodes are associated with a higher risk of subsequent adverse outcomes, such as cardiovascular disease, diabetes, cognitive deficits, and dementia, throughout a person's life span [10–13]. For example, Dotson et al. found that among adults aged ≥50 years, having one DS conferred an 87% increased risk of dementia risk compared with those without DS while having two or more episodes nearly doubled the risk [10]. Therefore, an improved understanding of what contributes to the occurrence and multiple DSs among the middle-aged and elderly populations is essential for promoting healthy aging.

The causes of DSs are complex and not completely understood. Previous studies have suggested that risk factors for DSs include being female, having low socioeconomic status, unemployment, cognitive impairment, chronic diseases, psychological trauma, and other stressful life events [14–16]. Developmental Origins of Health and Disease (DOHaD) theory posits that early life events can influence physical and mental health in adulthood [17, 18]. Adverse childhood experiences (ACEs) refer to a range of negative events occurring before the age of 18 years, such as physical, sexual, and emotional abuse, neglect, and parental loss [19]. Local and overseas evidence indicates that ACE is a risk factor for DSs [20–22]. In a cross-sectional analysis of Chinese adults, Yang et al. found that a single ACE experienced before the age of 17 years was associated with higher DS scores [23]. Similarly, a study from the US found that a high ACE score exposed from birth to age 18 years was associated with more DSs during adulthood [24]. The 2010 Behavioral Risk Factor Surveillance Survey of the US Center for Disease Control and Prevention found that older participants who reported multiple exposures to ACEs before the age of 18 years were more likely to have higher levels of DSs later in life [25]. However, previous work has only examined this association by either focusing on a single type of ACE in isolation without considering their high cooccurrence, or the overall risk based on the total number of ACEs by disregarding the potentially different physiological effects of various types of ACEs [26–28].

ACEs with different characteristics may produce diverse effects on health outcomes. In recent years, dividing complex ACEs into various dimensions with similar features has become a burgeoning research area. An increasing number of studies have emphasized the importance of deconstructing ACEs into threat-related and deprivation-related ACEs. The Dimensional Model of Adversity and Psychopathology (DMAP) [27] indicates that threat-related ACEs mostly involve harm or threat of harm, such as witnessing community violence, domestic violence, and physical abuse. Meanwhile, deprivation-related ACEs indicate the absence of expected input from the environment, such as poverty, neglect, and limited social stimulation. In accordance with DMAP, the two core dimensions may differ in underlying mechanisms regarding their risks for neurodevelopment and, ultimately, psychopathology or behavior problems. Threat-related ACEs may involve changes in the hippocampus, amygdala, and ventromedial prefrontal cortex structure, function, and coupling, resulting in disruptions in fear and emotion-learning processes and emotion management skills. Deprivation-related ACEs preferentially affect the structure of the associated cortex, causing disruptions in reward learning and cognitive processing capacity [27, 29–33]. Emerging studies have applied the DMAP approach to explore the dimension-specific effects of ACEs on DSs. A study of 247 youth aged 8-16 years in Seattle, US, found that threat, but not deprivation-related ACEs, were associated with advanced pubertal stage and accelerated DNA methylation (DNAm) age; and older DNAm age was related to more severe DSs [32]. In an HIV-positive adult sample, Clark et al. found that threat, but not deprivation exposure, was associated with neuropsychiatric symptoms [34]. However, a study conducted on 306 Latinx youth found that economic hardship (deprivation), but not immigration enforcement fear (threat), was positively associated with depression [35]. These studies on youths and adults from non-Chinese population provide cues that threat-related ACEs and deprivation-related ACEs may affect DSs differently. Apart from the different disease status, diet, and lifestyle [36–38], middle-aged and elderly Chinese face some unique challenges imposed by traditional Chinese culture, such as filial piety, that make the findings from other populations difficult to apply to them. For example, middle-aged and elderly Chinese are commonly responsible for taking care of their aging parents and supporting their adult children and may even be the primary caregiver of the third generation, while being the breadwinners of their families. In contrast, they frequently disregard their mental health, receive minimal support from family members, and lack access to related services, aggravating the challenges described above. Furthermore, whether threat-related and deprivation-related ACEs are specially associated with later-life DSs among Chinese is yet to be determined, hindering the precision prevention of depression. Latent class analysis (LCA) is a person-centered approach for identifying individuals who share similar characteristics, i.e., the so-called clusters [26]. LCA can be an alternative to objectively deconstructing ACEs into various dimensions, i.e., threat-related and deprivation-related ACEs. Kim et al. used LCA to identify latent clusters of ACEs and found that the “high adversity” and “child abuse” clusters, but not the “parental substance use” cluster, were significantly associated with major depression compared with the “low adversity” cluster [39]. Hence, simultaneously utilizing the two aforementioned methods of dealing with ACEs on the same individuals will be conducive to elucidating whether specific dimensions of ACEs affect DSs differently among middle-aged and elderly Chinese. To our knowledge, no study has yet investigated the associations between ACEs and DSs among the middle-aged and elderly Chinese population by using both the LCA and dimensional approach.

Given these gaps and the basis of longitudinal data from CHARLS, we aimed to explore the specific associations between the two dimensions of ACEs identified via LCA and the dimensional approach and the new occurrence of DSs and the number of times with DSs among the middle-aged and elderly Chinese population. We also performed sex-specific and age-specific analyses, considering sex and age differences in DSs [40, 41].

## 2. Methods

### 2.1. Study Design and Sample

This study used data from CHARLS, a prospective cohort that enrolled adults aged 45 years old and above from 450 urban communities and rural villages across China. CHARLS includes assessments of social, economic, and health statuses. The baseline survey for this study was conducted between 2011 and 2012, with respondents followed up every 2 years via face-to-face computer-assisted personal interview. All data in CHARLS are freely accessible to researchers worldwide (http://charls.pku.edu.cn/). The study protocol was approved by the ethical committees of Peking University and conformed to the ethical guidelines of the 1975 Declaration of Helsinki. Further details regarding CHARLS consent have been described previously [42].

**Figure 1:**
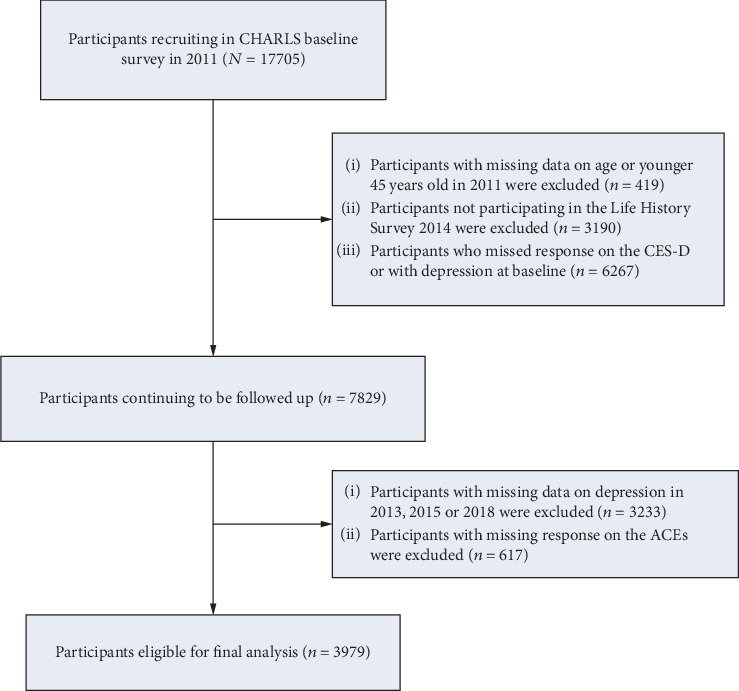
Flow chart of the study participant selection.

In the present study, we used four waves of the CHARLS survey, including the baseline conducted from 2011 to 2012 and three follow-ups from 2013 to 2018.  Figure 1 shows the flow chart of the selection of study participants. A total of 17705 participants joined the study and provided written informed consent at the baseline. We excluded participants with missing data on age or who were younger than 45 years at the time of recruitment (*n* = 419), did not participate in the Life History Survey in 2014 (*n* = 3190), had missing data on the 10-item Center for Epidemiological Studies Depression Scale (CES-D-10), or exhibited DSs at baseline (*n* = 6267). In addition, participants who had missing data on CES-D-10 during the follow-up from 2013 to 2018 (*n* = 3233) and missing responses on ACEs were also excluded (*n* = 617). Finally, the analysis included 3979 eligible participants. The baseline characteristics between included and excluded participants are compared in Supplementary Table S1.

### 2.2. Definition of ACEs

We measured ACEs by using the Life History Survey Questionnaire from 2014. Each participant self-reported their experiences on the following adversities before the age of 18 years: (1) unsafe community dwelling, (2) peer bullying, (3) female guardian physical abuse, (4) male guardian physical abuse, (5) being beaten by siblings, (6) parents frequently quarreling, (7) biological mother's absence, (8) biological father's absence, (9) food scarcity, (10) poor household economic conditions, (11) loneliness, (12) absence of care and attention from female guardian, (13) absence of love from female guardian, (14) mother being hit by father, and (15) father being hit by mother.

### 2.3. Handling ACEs

In accordance with the questionnaire results, one point was assigned for each ACE that the participants had experienced and zero point for those nonpresented corresponding ACEs. We handled ACEs in three ways. First, we generated a total ACE score by summing all ACE points. Therefore, the total ACE score that each participant received was a score out of 15. Second, we calculated two-dimensional ACE scores by classifying ACEs into threat-related and deprivation-related ACEs, in accordance with the characteristics of ACEs [27]. In this study, threat-related ACEs included unsafe community dwelling, peer bullying, female guardian physical abuse, male guardian physical abuse, being beaten by siblings, parents frequently quarreling, mother being hit by father, and father being hit by mother. Deprivation-related ACEs included biological mother's absence, biological father's absence, food scarcity, poor household economic conditions, loneliness, absence of care and attention from female guardian, and absence of love from female guardian. The range of threat-related and deprivation-related ACE scores was 0-8 and 0-7, respectively. To examine different thresholds of various ACE dimensions associated with the new occurrence of DSs or the number of times DSs were exhibited, we generated three categorical variables, namely, total, threat-related, and deprivation-related ACEs, in accordance with their respective ACE scores. Following a prior study on ACEs in CHARLS [43], participants were categorized into four groups (0 ACE, 1 ACE, 2 ACEs, and ≥3 ACEs) for analysis on the basis of cumulative scores. Third, we used LCA to identify latent clusters of the participants who shared similar patterns of ACEs by using Mplus version 7.

**Figure 2:**
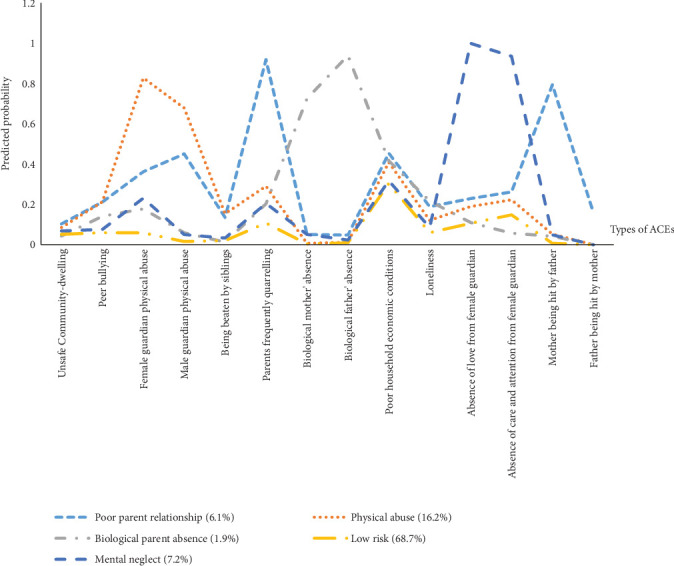
Profile plot for the five ACE clusters.

Table 1 and Figure 2 present the results of LCA. We compared models for two to seven classes and selected the best-fitting class solution on the basis of model fit indices of the Akaike information criteria (AIC), Bayesian information criteria (BIC), sample size-adjusted BIC (ABIC), and entropy value. Lower values of AIC, BIC, and ABIC indicate better fit, while an entropy value that approaches one implies better distinction of classes [44]. All participants were assigned to their best-fit cluster on the basis of the optimal class solution, creating a new categorical variable, i.e., the ACE cluster, for subsequent analyses. Ultimately, we selected a model with five ACEs: the “low risk (LR)” cluster (68.7%), the “poor parent relationship (PPR)” cluster (6.1%), the “physical abuse (PA)” cluster (16.2%), the “biological parent absence (BPA)” cluster (1.9%), and the “mental neglect (MN)” cluster (7.2%).

### 2.4. Definition of DSs

DSs were examined via CES-D-10, a well-accepted and widely used self-rating scale in the Chinese population with good internal consistency and test-retest reliability. For example, in a community sample of older adults in Hong Kong, Cronbach's *α* was 0.78 and test-retest *r* was 0.44 [45]. The results of a confirmatory factor analysis indicated adequate reliability and validity in CHARLS [46]. Internal consistency in this study was satisfactory (Cronbach's *α* = 0.84). The 10 items of CES-D-10 were rated as either positive or negative. The scale includes “rarely (<1 day per week),” “some days (1–2 days per week),” “occasionally (3–4 days per week),” and “most (5–7 days per week)” for each item. Moreover, “rarely,” “some days,” “occasionally,” and “most” are scored as 0, 1, 2, and 3 for negative items and 3, 2, 1, and 0 for positive ones, respectively. The sum of the scores for the 10 items generates an overall score (range: 0–30). An overall score that is equal to or more than 10 indicates having DSs [47]. In the final analysis, two ways were used to handle future DSs. First, the participants were defined as experiencing a new occurrence of DSs if their CES-D-10 scores were equal to or larger than 10 [47] during any follow-up. Then, we recorded how many times the participants' CES-D-10 scores were equal to or greater than 10 acorss the three follow-ups and categorized them into four groups based on the number of times that DSs were detected, i.e., 0, 1, 2, and 3.

### 2.5. Other Variables

Various demographic factors were collected at baseline, including sex (male and female); age in years; educational level (categorized as elementary school or below, middle/high/vocational school, and equal or more than an associate degree); marital status (married, separated, and never married/divorced/widowed); hukou (representing the household registration system used in Mainland China, categorized as agricultural, nonagricultural, and unified residence); and annual household income (<27600 RMB and ≥27600 RMB, grouped by yearly median income). Additionally, behavioral factors were assessed, such as smoking status (nonsmoker, ex-smoker, and smoker), and drinking status (categorized as ≥1 time/month, <1 time/month and nondrinker). Health status indicators included chronic diseases status (yes and no), life satisfaction (classified as satisfied, somewhat satisfied, and dissatisfied), and CES-D-10 score at baseline (the score of the CES-D-10 assessed in 2011).

### 2.6. Statistical Analysis

We described participant characteristics at baseline in accordance with the incidence of DSs. Data were presented as mean and standard deviation (SD) for continuous variables and percentages for categorical variables. If appropriate, then the *t*-test or chi-squared test was performed to explore differences in baseline characteristics. The Cox proportional hazards regression models were utilized to examine the associations between ACEs and new occurrence of DSs. Ordered logistic regression models were adopted to explore the associations between ACEs and the number of times with DSs. We also assessed the linear trend across the three types of ACEs (total, threat-related, and deprivation-related ACEs). We performed stratified analyses via prespecified baseline subgroups of age and sex. LCA was conducted using Mplus version 7 to identify latent clusters of ACEs. For the variables to be adjusted, we first used a directed acyclic graph (DAG) (Supplementary Figure S1) to identify the minimum set of potential confounders associated with ACEs and the new occurrence of DSs or the number of times with DSs during follow-up. The variables selected based on DAG were age, sex, and hukou. We also included those variables in the final regression models if they were associated (*P* < 0.05) with DSs in a univariate model or previously reported to be significant [48, 49]. All other statistical analyses were conducted using R version 4.1.1 for Window, and a two-tailed *P* < 0.05 was considered statistically significant.

## 3. Results

### 3.1. Participant Characteristics

Table 2 provides the baseline characteristics of the study participants in accordance with the new occurrence of DSs. Briefly, this study consisted of 3979 participants, including 2119 (53.3%) males and 1856 (46.6%) females, with a mean age of 56.5 years. During the follow-up from 2011 to 2018, 1656 participants with a new occurrence of DSs were documented. Among those with DSs, 998 (60.3%), 438 (26.4%), and 220 (13.3%) exhibited DSs one, two, and three times, respectively. Overall, most participants (90.1%) reported experiencing at least one type of ACE in childhood, while 41.3% reported experiencing three or more ACEs. Nearly half of the participants (46.5%) reported at least one type of threat-related ACEs, while 14.6% reported three or more threat-related ACEs. More than three-fourths of the participants (83.9%) indicated at least one form of deprivation-related ACEs, and 17.7% had three or more deprivation-related ACEs. Participants who experienced a new occurrence of DSs had lower educational level and household income, but higher proportion of agricultural hukou. They also tended to be women, nonsmokers, nondrinkers, diagnosed with a chronic disease, and less satisfied with life and have higher total, threat-related, and deprivation-related ACE scores. Moreover, the proportions that belonged to the “PPR” and the “PA” clusters were higher among the participants who experienced new occurrence of DSs (all *P* values < 0.05).

### 3.2. Associations between ACEs and New Occurrence of DSs

**Figure 3:**
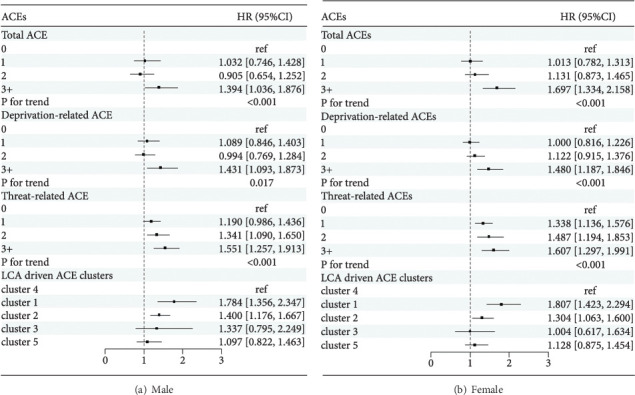
The associations between ACEs and the new occurrence of DSs according to sex in model 2. Cluster 1: poor parent relationship; cluster 2: physical abuse; cluster 3: biological parent absence; cluster 4: low risk (reference); cluster 5: mental neglect. HR (95% CI): hazard ratio (95% confidence interval). Model 2 is adjusted for deprivation-related ACEs (if applicable), threat-related ACEs (if applicable), age, marital status, education level, hukou, chronic disease, smoking status, drinking status, life satisfaction, household income, and CES-D-10 score at baseline.

**Figure 4:**
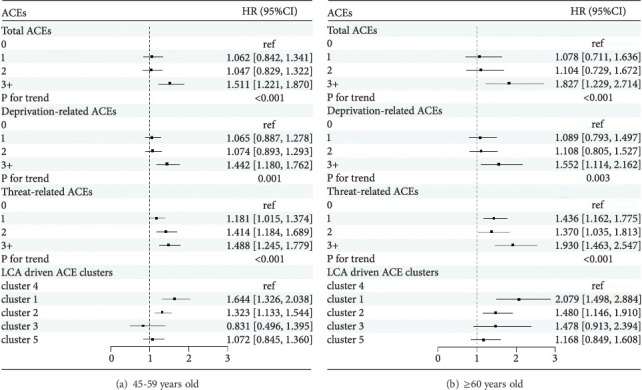
The associations between ACEs and new occurrence of DSs according to age in model 2. Cluster 1: poor parent relationship; cluster 2: physical abuse; cluster 3: biological parent absence; cluster 4: low risk (reference); cluster 5: mental neglect. HR (95% CI): hazard ratio (95% confidence interval). Model 2 was adjusted for deprivation-related ACEs (if applicable), threat-related ACEs (if applicable), sex, marital status, education level, hukou, chronic disease, smoking status, drinking status, life satisfaction, household income, and CES-D-10 score at baseline.

Table 3 presents the associations between ACEs and the new occurrence of DSs. In the adjusted model 2, higher scores of total, deprivation-related, and threat-related ACEs were positively associated with the new occurrence of DSs (*P*_total⁣ACEs_ for trend < 0.001, *P*_deprivation−related⁣ACEs_ for trend < 0.001, and *P*_threat−related⁣ACEs_ for trend < 0.001). For total and deprivation-related ACEs, only the ≥3 ACE group was significantly associated with the new occurrence of DSs when compared with the 0 ACE group. The multivariate-adjusted hazard ratios (HRs) (95%confidence interval (CI)) were 1.562 (1.296, 1.882) and 1.446 (1.221, 1.712), respectively. For the threat-related ACEs, all three groups (1 ACE, 2 ACEs, and ≥3 ACEs) were significantly associated with the new occurrence of DSs, with HRs (95% CI) of 1.260 (1.115, 1.425), 1.407 (1.212, 1.634), and 1.585 (1.366, 1.840), respectively. For the LCA-derived clusters of ACEs, compared with the “LR” cluster, the “PPR” and “PA” clusters were associated with the new occurrence of DSs, with HRs (95% CI) of 1.758 (1.471, 2.100) and 1.361 (1.194, 1.553), respectively. Subgroup analysis by sex and age also indicated that threat-related ACEs were more detrimental to DSs than total and deprivation-related ACEs, with the results presented in Figures 3 and 4, respectively. Sensitivity analyses were performed by further adjusting chronic disease status in 2018 and excluding baseline life satisfaction, respectively, and the results remained unchanged (Supplementary Table S2–7).

### 3.3. Associations between ACEs and the Number of Times with DSs

**Figure 5:**
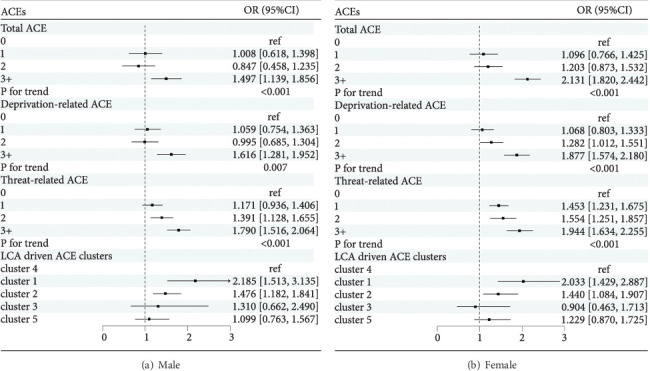
The associations between ACEs and number of times with DSs according to sex in the model 2. Cluster 1: poor parent relationship; cluster 2: physical abuse; cluster 3: biological parent absence; cluster 4: low risk (reference); cluster 5: mental neglect. OR (95% CI): odds ratio (95% confidence interval). The model 2 was adjusted for deprivation-related ACEs (if applicable), threat-related ACEs (if applicable), age, marital status, education level, hukou, chronic disease, smoking status, drinking status, life satisfaction, household income, and CES-D-10 score at baseline.

**Figure 6:**
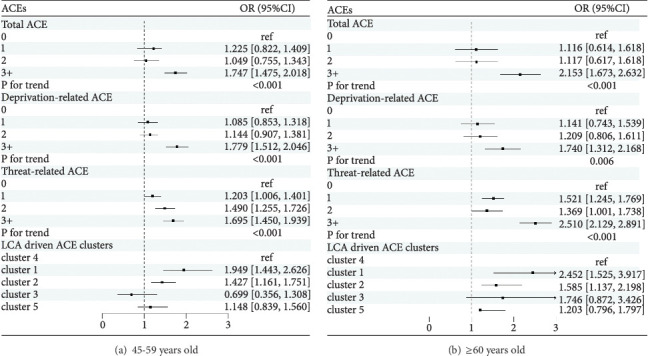
The associations between ACEs and number of times with DSs according to age in model 2. Cluster 1: poor parent relationship; cluster 2: physical abuse; cluster 3: biological parent absence; cluster 4: low risk (reference); cluster 5: mental neglect. OR (95% CI): odds ratio (95% confidence interval). The mode1 2 was adjusted for deprivation-related ACEs (if applicable), threat-related ACEs (if applicable), sex, marital status, education level, hukou, chronic disease, smoking status, drinking status, life satisfaction, household income, and CES-D-10 score at baseline.

Table 4 presents the associations between ACEs and the number of times with DSs. In the adjusted model 2, higher ACE scores were positively associated with an increased number of times with DSs, regardless of ACE types (all *P* for trend < 0.001). In terms of total and deprivation-related ACEs, the risk of an increased number of times with DSs was only associated with the ≥3 ACE group when compared with the 0 ACE group. The multivariate-adjusted odds ratios (ORs) (95% CI) of the number of times with DSs for total and deprivation-related ACEs among the ≥3 ACE group were 1.818 (1.441, 2.303) and 1.731 (1.386, 2.164), respectively. For threat-related ACEs, all three groups were significant and the adjusted ORs (95% CI) were 1.305 (1.112, 1.532) for the 1 ACE group, 1.451 (1.191, 1.766) for the 2 ACE group, and 1.871 (1.525, 2.294) for the ≥3 ACE groups. For the LCA-derived clusters of ACEs, compared with the “LR” cluster, only the “PPR” and “PA” clusters were associated with multiple detected DSs, with ORs (95% CI) of 2.075 (1.610, 2.669) and 1.443 (1.212, 1.718), respectively. The results of subgroup analysis by sex and age were consistent with the total population. The results are shown in Figures 5 and 6, respectively. To assess the robustness of the main findings, sensitivity analyses were performed by making additional adjustments to chronic disease status in 2018 and excluding baseline life satisfaction, and the results remain unchanged. The detailed information is shown in Supplementary Tables S8–13.

## 4. Discussion

To our knowledge, this study is the first to investigate the longitudinal associations between different dimensions of ACEs and DSs among the middle-aged and elderly Chinese population, using the dimensional approach and LCA. ACEs were found to be prevalent among middle-aged and elderly Chinese, with 90.1% of the participants reported at least one type of ACEs before the age of 18 years. Furthermore, nearly half (46.5%) reported at least one type of threat-related ACEs, and over three quarters (83.9%) reported at least one form of deprivation-related ACEs. Given the negative health effects of ACEs and aging population, integrating ACE screening into regular health checks for middle-aged and elderly individuals is recommended. Targeted mental health interventions should also be delivered to those who experienced multiple adversities in their early life, particularly those exposed to threat-related ACEs, in order to promote healthy aging.

The results of our study suggest that higher scores for the total, deprivation-related, and threat-related ACEs were all positively associated with higher risks of new occurrence of DSs and multiple detected DSs in mid to late life, with a stronger association observed in threat-related ACEs. In LCA, compared with the “LR” cluster, the “PPR” and “PA” clusters, but not the “BA” and “MN” clusters, were associated with an increased risk of incident DSs. The results support the hypothesis that threat-related ACEs are more prone to produce a toxic effect on DSs. Previous studies on the associations between ACEs and DSs have focused on prevailing approaches for handling ACEs, i.e., either focusing on a single type of ACEs [23, 50] or based on the total number of ACEs [51–53], which can hinder further insight into ACEs's effects on DSs. A deep understanding of whether a specific ACE exerts a more toxic effect is the key to developing target interventions for preventing DSs in mid to late life, because ACEs with different characteristics tend to have diverse effects on health. Nevertheless, existing findings lack information regarding which specific ACE domains largely contribute to DSs. Recently, dividing complex ACEs into various dimensions with common features, particularly threat-related and deprivation-related ACEs, is considered an alternative method for identifying specific dimensions of ACEs that influence physical and mental health [27]. Nonetheless, limited studies have simultaneously explored the associations of DSs with threat-related and deprivation-related ACEs in the middle-aged and elderly Chinese population. Here, we found a stronger association between threat-related ACEs and DSs than between deprivation-related and total ACEs. Our finding is consistent with the results of the Avon Longitudinal Study of Parents and Children, which indicated that sexual abuse and adversities that involved physical and emotional threats, particularly during adolescence, exhibited more prominent associations with DSs in young adulthood than other ACEs [54]. In this study, we also applied LCA to identify different ACE clusters more objectively. Our results indicated that certain types of ACE clusters, such as the “PPR” and the “PA” clusters, are significant predictors of future DSs among middle-aged and elderly Chinese, supporting the finding that threat-related ACEs exhibit a stronger association with DSs.

The mechanisms that explain why threat-related ACEs represent stronger associations with DS are still incompletely precise. Pubertal onset timing may partly explain the obtained results. Researchers have found that exposure to threat-related ACEs, but not to deprivation-related ACEs, accelerates pubertal onset [32, 55], and earlier pubertal onset is associated with a greater risk for depression [56–59]. Alterations in the physiological stress response systems, such as the hypothalamic-pituitary-adrenal (HPA) axis and the hypothalamic-pituitary-gonadal (HPG) axis, may play a role in the associations between threat-related ACEs and pubertal timing [60]. Another possible mechanism is DNAm age. Sumner et al. found that threat-related ACEs are associated with accelerated DNAm, and DNAm age is a mediator between threat exposure and DSs [32]. Epigenetic aging is a source of inflammatory signaling, particularly in senescent cells [61]. Studies have found that adults exposed to child abuse have steeper inflammatory trajectories [62], and systemic inflammation is direct with increased DSs [63]. This evidence indicates that inflammation may be one critical pathway through which epigenetic effects of ACEs on DSs persist into the middle and later life. However, further research is warranted to validate the above hypothesis. The present study also found that higher scores of total, deprivation-related, and threat-related ACEs were associated with a higher frequency of exhibiting DSs. Our results indicated that ACEs might predispose middle-aged and elderly adults to resistant DSs. A longitudinal study further supported these findings, as it found a positive association between the number of childhood emotional and sexual abuse perpetrations, and the occurrence of depressive episodes in adulthood [64]. A systematic review and meta-synthesis suggested that those with a history of childhood maltreatment face greater odds of multiple DSs than those without such experience [65]. However, studies on ACEs with multiple DSs are scarce. Inspired by a meta-analysis that demonstrated an association between childhood maltreatment and poor response during treatment for DSs [66], we formulated a hypothesis that increased exposure to ACEs might also lead to more unsatisfactory treatment response and contributes to a higher risk of experiencing multiple DSs. Moreover, patients with depression commonly experience self-stigma [67], which can further hinder their treatment-seeking behavior [68]. A longer duration of untreated DSs may enhance one's vulnerability to multiple DSs.

## 5. Strengths and Limitations

Our study has several strengths. First, we used longitudinal data from CHARLS, a nationally representative survey, to examine the associations between ACEs and DSs. Second, we investigated associations of various dimensions of ACEs with DSs by using LCA and the ACE core dimension strategy (deprivation and threat). The consistency of the findings from both approaches suggests the robustness of the results. Our study extends existing evidence on dividing ACEs into specific dimensions to explore their varying effects on DSs among middle-aged and elderly Chinese. Third, our results revealed that experiencing multiple ACE exposures increased the risk of exhibiting DSs multiple times, implying its utility for the risk stratification of DSs among the middle-aged and elderly. Researchers and health personnel should work together to identify potential groups with higher risks of DSs among the middle-aged and elderly populations and then develop target prevention programs to ensure their healthy aging.

However, this study also has several limitations. First, the use of the retrospective reporting of ACEs may bear some recall bias. Another limitation is that the ACEs used in the present study are one-time measurements that may not reflect participants' overall experience before the age of 18 years because individuals are likely to experience various types of ACEs across different age stages in different settings [69]. Furthermore, the specific types and duration of ACEs, and the timing of ACE exposure, may affect development in myriad ways, exerting other effects, or triggering a different response at various life stages [21]. In the current study, no data on the specific ages in which each adversity occurred were available. Therefore, we could not further explore the effects of ACEs occurring at different age stages (the critical windows of physical and mental health development) and with different durations. Moreover, confounders or moderators, including participants' birth weight and gestational age at birth [21] as well as current stressors, may interact with ACEs and affect the associations of ACEs with the new occurrence of DSs and the detection of multiple DSs. Unfortunately, these data are lacking in CHARLS. Growing evidence indicates that protective factors, such as resilience [70] and supportive family environments [71], can buffer the adverse effects of ACEs. Future studies should also prioritize investigating variables that act as protective factors against ACE-associated DSs. In addition, some background variables differ between included and excluded participants, which may produce selection bias. To address these gaps, birth cohorts with rigorous design and a comprehensive assessment of variables could be instrumental in prospectively collecting individuals' ACEs from the prenatal period to adolescence and eventually unpacking the associations between ACEs occurring in early life and mid–late life DSs.

## 6. Conclusions

Higher ACE scores in early life predict higher risks of new occurrence of DSs and an increased number of times DSs are reported in mid–late life. Our study also provides preliminary evidence on the effects of different dimensions of ACEs on DSs. The two-core dimension strategy and the LCA approach cross-validate that threat-related ACEs are riskier than total and deprivation-related ACEs for DSs, highlighting the importance of deconstructing ACEs in order to illuminate their distinct role in DSs as well as the underlying mechanisms for designing targeted interventions.

## Figures and Tables

**Table 1:** Model fit statistics and latent classes of the participants.

Models	*K*	log(*L*)	AIC	BIC	ABIC	Entropy	LMR	BLRT	Population proportion (%)
2	29	-18075.946	36209.89	36392.267	36300.118	0.717	0.0000	0.0000	24.8/75.2
3	44	-17880.48	35848.97	36125.678	35985.866	0.804	0.0013	0.0000	6.9/15.6/75.5
4	59	-17718.015	35554.03	35925.068	35737.593	0.847	0.0000	0.0000	18.0/1.9/74.0/6.1
5⁣^∗^	74	-17570.456	35288.91	35754.283	35519.144	0.849	0.0141	0.0000	6.1/16.2/1.9/68.7/7.2
6	89	-17498.752	35175.5	35735.206	35452.404	0.783	0.0000	0.0000	7.2/13.4/62.8/2.7/7.9/6.0
7	104	-17449.808	35107.62	35761.649	35431.183	0.700	0.4000	0.0000	1.8/5.5/6.8/2.3/12.9/7.0/63.8

The symbol ‘⁣^∗^' indicates the best-fitting class solution.

**Table 2:** Baseline participant characteristics according to future depressive symptoms.

Variables	Characteristic	Overall (*N* = 3979), *n* (%)	Depression symptoms during follow-up		*P* value
			No depression (*n* = 2323), *n* (%)	Depression (*n* = 1656), *n* (%)	
Age (mean (SD))	—	56.48 (7.85)	56.57 (7.92)	56.37 (7.74)	0.439
Sex	Male	2119 (53.3)	1370 (59.0)	749 (45.2)	<0.001
	Female	1856 (46.6)	953 (41.0)	903 (54.5)	
Unsafe community dwelling	Yes	245 (6.2)	119 (5.1)	126 (7.6)	0.002
Peer bullying	Yes	394 (9.9)	185 (8.0)	209 (12.6)	<0.001
Female guardian physical abuse	Yes	889 (22.3)	462 (19.9)	427 (25.8)	<0.001
Male guardian physical abuse	Yes	640 (16.1)	350 (5.1)	290 (17.5)	0.043
Being beaten by siblings	Yes	204 (5.1)	98 (4.2)	106 (6.4)	0.003
Parents often quarreling	Yes	803 (20.2)	406 (17.5)	397 (24.0)	<0.001
Biological mother's absence	Yes	121 (3.0)	64 (2.8)	57 (3.4)	0.250
Biological father's absence	Yes	138 (3.5)	81 (3.5)	57 (3.4)	1.000
Food scarcity	Yes	2713 (68.2)	1527 (65.7)	1186 (71.6)	<0.001
Poor household economic condition	Yes	1335 (33.6)	705 (30.3)	630 (38.0)	<0.001
Loneliness	Yes	335 (8.4)	142 (6.1)	193 (11.7)	<0.001
Absence of watching over from female guardian	Yes	861 (21.6)	472 (20.3)	389 (23.5)	0.018
Absence of love from female guardian	Yes	738 (18.5)	423 (18.2)	315 (19.0)	0.543
Mother being hit by the father	Yes	282 (7.1)	130 (5.6)	152 (9.2)	<0.001
Father being hit by the mother	Yes	44 (1.1)	19 (0.8)	25 (1.5)	0.057
Age group (years)	45-59	2640 (66.3)	1519 (65.4)	1121 (67.7)	0.138
	≥60	1339 (33.7)	804 (34.6)	535 (32.3)	
Marital status	Married	3576 (89.9)	2102 (90.5)	1474 (89.0)	0.187
	Separated	148 (3.8)	86 (2.2)	62 (1.6)	
	Never married/divorced/widowed/	255 (6.4)	135 (3.4)	120 (3.0)	
Education	Elementary school or below	2208 (55.5)	1165 (50.2)	1043 (63.0)	<0.001
	Middle/high/vocational school	1674 (42.1)	1076 (46.3)	598 (36.1)	
	Equal or more than associate degree	96 (2.4)	82 (3.5)	14 (0.8)	
Hukou	Agricultural	3077 (77.4)	1703 (73.3)	1374 (83.0)	<0.001
	Nonagricultural	867 (21.8)	596 (25.7)	271 (16.4)	
	Unified residence	34 (0.9)	23 (1.0)	11 (0.7)	
Chronic disease	No	1558 (39.2)	999 (43.0)	559 (33.8)	<0.001
	Yes	2378 (59.8)	1299 (55.9)	1079 (65.2)	
Smoking status	Nonsmoker	2316 (58.2)	1287 (55.4)	1029 (62.1)	<0.001
	Ex-smoker	332 (8.3)	201 (8.7)	131 (7.9)	
	Smoker	1331 (33.5)	835 (35.9)	496 (30.0)	
Drinking status	≥1 time/month	1164 (29.3)	741 (31.9)	423 (25.5)	<0.001
	<1 time/month	339 (8.5)	208 (9.0)	131 (7.9)	
	Nondrinker	2476 (62.2)	1374 (59.1)	1102 (66.5)	
Life satisfaction	Satisfied	1006 (27.0)	664 (30.0)	342 (22.6)	<0.001
	Somewhat satisfied	2458 (65.9)	1447 (65.3)	1011 (66.8)	
	Dissatisfied	264 (7.1)	104 (4.7)	160 (10.6)	
Household income (yuan/year)	<27600	1983 (50)	1082 (46.7)	901 (54.6)	<0.001
	≥27600	1984 (50)	1234 (53.3)	750 (45.4)	
Total ACE score	0	394 (9.9)	262 (11.3)	132 (8.0)	<0.001
	1	961 (24.2)	607 (26.1)	354 (21.4)	
	2	979 (24.6)	616 (26.5)	363 (21.9)	
	≥3	1645 (41.3)	838 (36.1)	807 (48.7)	
Threat-related ACE score (%)	0	2129 (53.5)	1341 (57.7)	788 (47.6)	<0.001
	1	892 (22.4)	499 (21.5)	393 (23.7)	
	2	508 (12.8)	274 (11.8)	234 (14.1)	
	≥3	450 (11.3)	209 (9.0)	241 (14.6)	
Deprivation-related ACE score (%)	0	640 (16.1)	410 (17.6)	230 (13.9)	<0.001
	1	1380 (34.7)	839 (36.1)	541 (32.7)	
	2	1256 (31.6)	749 (32.2)	507 (30.6)	
	≥3	703 (17.7)	325 (14.0)	378 (22.8)	
LCA-driven ACE cluster	Low risk	2733 (68.7)	1673 (72.0)	1060 (64.0)	<0.001
	Poor parent relationship	241 (6.1)	102 (14.8)	139 (18.2)	
	Physical abuse	644 (16.2)	343 (4.4)	301 (8.3)	
	Biological parent absence	76 (11.9)	43 (1.9)	33 (2.0)	
	Mental neglect	287 (7.2)	162 (7.0)	123 (7.4)	

Note: ACEs: adverse childhood experiences. “Yes” represents “experienced a specific ACE.” Concerning the length of the table, we did not include the prevalence of “without experience of the specific ACE,” which should be 100%—% of yes. The *P* values indicate if there are significant differences in the distribution of ACE types and baseline characteristics between the nondepression and depression groups.

**Table 3:** Associations between ACEs and the new occurrence of DSs in the Cox proportional hazards model.

	Crude model		Adjusted model 1		Adjusted model 2	
	HR (95% CI)	*P* value	HR (95% CI)	*P* value	HR (95% CI)	*P* value
Total ACE score						
0	Ref		Ref^a^		Ref^a1^	
1	1.159 (0.949, 1.415)	0.149	1.090 (0.891, 1.333)	0.404	1.040 (0.850, 1.273)	0.701
2	1.161 (0.951, 1.416)	0.143	1.099 (0.899, 1.344)	0.357	1.042 (0.851, 1.274)	0.692
≥3	1.712 (1.424, 2.058)	<0.001	1.664 (1.382, 2.005)	<0.001	1.562 (1.296, 1.882)	<0.001
*P* for trend	<0.001		<0.001		<0.001	
Deprivation-related ACE score						
0	Ref		Ref^b^		Ref^b1^	
1	1.140 (0.977, 1.330)	0.095	1.089 (0.925, 1.283)	0.306	1.050 (0.897, 1.228)	0.545
2	1.188 (1.017, 1.389)	0.030	1.117 (0.947, 1.319)	0.190	1.060 (0.904, 1.242)	0.474
≥3	1.809 (1.535, 2.131)	<0.001	1.519 (1.273, 1.814)	<0.001	1.446 (1.221, 1.712)	<0.001
*P* for trend	<0.001		<0.001		<0.001	
Threat-related ACE score						
0	Ref		Ref^c^		Ref^c1^	
1	1.272 (1.127, 1.436)	<0.001	1.239 (1.089, 1.410)	0.001	1.260 (1.115, 1.425)	<0.001
2	1.342 (1.160, 1.553)	<0.001	1.442 (1.236, 1.681)	<0.001	1.407 (1.212, 1.634)	<0.001
≥3	1.688 (1.461, 1.950)	<0.001	1.572 (1.343, 1.841)	<0.001	1.585 (1.366, 1.840)	<0.001
*P* for trend	<0.001		<0.001		<0.001	
LCA-driven ACE						
Low risk	Ref		Ref^a^		Ref^a1^	
Poor parent relationship	1.788 (1.498, 2.134)	<0.001	1.797 (1.504, 2.148)	<0.001	1.758 (1.471, 2.100)	<0.001
Physical abuse	1.298 (1.142, 1.475)	<0.001	1.395 (1.224, 1.590)	<0.001	1.361 (1.194, 1.553)	<0.001
Biological parent absent	1.207 (0.854, 1.707)	0.287	1.067 (0.754, 1.510)	0.715	1.108 (0.782, 1.571)	0.563
Mental neglect	1.146 (0.951, 1.381)	0.153	1.098 (0.908, 1.327)	0.334	1.100 (0.910, 1.330)	0.325

^a^Adjusted for age, sex, marital status, education level, hukou, chronic disease, smoking status, drinking status, life satisfaction, and household income. ^a1^Additionally adjusted for CES-D-10 score at baseline based on model 1. ^b^Adjusted for threat-related ACE score, age, sex, marital status, education level, hukou, chronic disease, smoking status, drinking status, life satisfaction, and household income. ^b1^Additionally adjusted for CES-D-10 scores at baseline based on model 1. ^c^Adjusted for deprivation-related ACE score, age, sex, marital status, education level, hukou, chronic disease, smoking status, drinking status, life satisfaction, and household income. ^c1^Additionally adjusted for CES-D-10 scores at baseline based on model 1. ACEs: adverse childhood experiences; HR: hazard ratios; CI: confidence interval.

**Table 4:** Associations between ACEs and the number of times with DSs in ordered logistic regression model.

	Crude model		Adjusted model 1		Adjusted model 2	
	OR (95% CI)	*P* value	OR (95% CI)	*P* value	OR (95% CI)	*P* value
Total ACE score						
0	Ref		Ref^a^		Ref^a1^	
1	1.226 (0.965, 1.562)	0.097	1.130 (0.882, 1.452)	0.337	1.089 (0.848, 1.404)	0.505
2	1.209 (0.953, 1.539)	0.121	1.102 (0.861, 1.416)	0.444	1.049 (0.817, 1.351)	0.709
≥3	2.032 (1.628, 2.548)	<0.001	1.932 (1.535, 2.444)	<0.001	1.818 (1.441, 2.303)	<0.001
*P* for trend	<0.001		<0.001		<0.001	
Deprivation-related ACE score						
0	Ref		Ref^b^		Ref^b1^	
1	1.192 (0.988, 1.441)	0.067	1.105 (0.908, 1.347)	0.320	1.081 (0.887, 1.320)	0.443
2	1.292 (1.068, 1.565)	0.009	1.161 (0.951, 1.419)	0.144	1.139 (0.931, 1.394)	0.207
≥3	2.276 (1.847, 2.808)	<0.001	1.829 (1.468, 2.283)	<0.001	1.731 (1.386, 2.164)	<0.001
*P* for trend	<0.001		<0.001		<0.001	
Threat-related ACE score						
0	Ref		Ref^c^		Ref^c1^	
1	1.344 (1.153, 1.566)	<0.001	1.328 (1.133, 1.555)	<0.001	1.305 (1.112, 1.532)	0.001
2	1.409 (1.168, 1.696)	<0.001	1.510 (1.241, 1.834)	<0.001	1.451 (1.191, 1.766)	<0.001
≥3	2.030 (1.672, 2.462)	<0.001	1.950 (1.592, 2.386)	<0.001	1.871 (1.525, 2.294)	<0.001
*P* for trend	<0.001		<0.001		<0.001	
LCA-driven ACE						
Low risk (LR)	Ref		Ref^a^		Ref^a1^	
Poor parent relationship (PPR)	2.207 (1.726, 2.817)	<0.001	2.157 (1.680, 2.763)	<0.001	2.075 (1.610, 2.669)	<0.001
Physical abuse (PA)	1.380 (1.169, 1.627)	<0.001	1.544 (1.301, 1.832)	<0.001	1.443 (1.212, 1.718)	<0.001
Biological parent absence (BPA)	1.213 (0.773, 1.873)	0.391	1.073 (0.675, 1.679)	0.760	1.028 (0.642, 1.619)	0.906
Mental neglect (MN)	1.177 (0.927, 1.489)	0.178	1.168 (0.914, 1.487)	0.209	1.145 (0.892, 1.464)	0.283

^a^Adjusted for age, sex, marital status, education level, hukou, chronic disease, smoking status, drinking status, life satisfaction, and household income. ^a1^Additionally adjusted for CES-D-10 score at baseline based on model 1. ^b^Adjusted for threat-related ACE score, age, sex, marital status, education level, hukou, chronic disease, smoking status, drinking status, life satisfaction, and household income. ^b1^Additionally adjusted for CES-D-10 scores at baseline based on model 1. ^c^Adjusted for deprivation-related ACE score, age, sex, marital status, education level, hukou, chronic disease, smoking status, drinking status, life satisfaction, and household income. ^c1^Additionally adjusted for CES-D-10 scores at baseline based on model 1. ACEs: adverse childhood experiences; OR: odds ratios; CI: confidence interval.

## Data Availability

All data are freely available from the website of CHARLS (http://charls.pku.edu.cn/).

## Abbreviations

ACEs: Adverse childhood experiences
DS: Depressive symptoms
LCA: Latent class analysis
CHARLS: The China Health and Retirement Longitudinal Study
DOHaD: The Developmental Origins of Health and Disease
BRFSS: Behavioral Risk Factor Surveillance Survey
CES-D-10: The 10-item Center for Epidemiological Studies Depression Scale
AIC: Akaike's information criteria
BIC: Bayesian information criteria
ABIC: Sample size-adjusted Bayesian information criteria
LR: Low risk
PPR: Poor parent relationship
PA: Physical abuse
BPA: Biological parent absent
MN: Mental neglect
HRs: Hazard ratios
CI: Confidence interval
ORs: Odds ratios
HPA: The hypothalamic-pituitary-adrenal axis
HPG: The hypothalamic-pituitary-gonadal axis
DNAm: DNA methylation
DAG: Directed acyclic graph.

## Supplementary Materials

Supplementary Materials Supplementary Figure S1 presents the directed acyclic graph for the association between ACEs and new occurrence of DSs or number of times participants reporting DSs. Supplementary Table S1 shows the baseline characteristics between included and excluded participants. The results of sensitivity analyses of the association between ACEs and new occurrence of DSs by making additional adjustment to the chronic disease status in 2018 are provided in Supplementary Tables S2-4. Supplementary Tables S5-7 show the results of sensitivity analyses of the association between ACEs and new occurrence of DSs by excluding baseline life satisfaction from the variables to be adjusted. Supplementary Tables S8-10 present the results of sensitivity analyses by further adjusting chronic disease status in 2018 on the association between ACEs and the number of times with DSs. Supplementary Tables S11-13 show the results of sensitivity analyses of the association between ACEs and the number of times with DSs by excluding baseline life satisfaction from the variables to be adjusted.
