# Supplementary material for: Unpacking the Specific Associations Between Adverse Childhood Experiences and Depressive Symptoms among the Middle-Aged and Elderly Chinese Populations: A Dimensional Approach and Latent Class Analysis in a Cohort Study
Source: Depress Anxiety. 2023 Aug 2;2023:8439527. doi: 10.1155/2023/8439527 (PMC11921828; doi:10.1155/2023/8439527)
Supplement: Supplementary Materials — Supplementary Figure S1 presents the directed acyclic graph for the association between ACEs and new occurrence of DSs or number of times participants reporting DSs. Supplementary Table S1 shows the baseline characteristics between included and excluded participants. The results of sensitivity analyses of the association between ACEs and new occurrence of DSs by making additional adjustment to the chronic disease status in 2018 are provided in Supplementary Tables S2-4. Supplementary Tables S5-7 show the results of sensitivity analyses of the association between ACEs and new occurrence of DSs by excluding baseline life satisfaction from the variables to be adjusted. Supplementary Tables S8-10 present the results of sensitivity analyses by further adjusting chronic disease status in 2018 on the association between ACEs and the number of times with DSs. Supplementary Tables S11-13 show the results of sensitivity analyses of the association between ACEs and the number of times with DSs by excluding baseline life satisfaction from the variables to be adjusted. [file 8439527.f1.docx]

**Supplementary materials**

**Title: Unpacking the specific associations between adverse childhood experiences and depressive symptoms among the middle-aged and elderly Chinese populations: A dimensional approach and latent class analysis in a cohort study**

Mengna Wei ^1^, Miyuan Wang ^1^, Rui Chang ^1^, Chunan Li ^1^, Ke Xu ^1^, Yanfen Jiang ^1^, Yimin Wang ^1^, Paiziyeti•Tuerxun ^1^, Jianduan Zhang ^1,2^.

Affiliations: ^1^ Department of Maternal and Child Health, School of Public Health, Tongji Medical College, Huazhong University of Science and Technology, Wuhan, China.

^2^ Key Laboratory of Environment and Health, Ministry of Education & Ministry of Environmental Protection, and State Key Laboratory of Environmental Health (Incubation), School of Public Health, Tongji Medical College, Huazhong University of Science and Technology, Wuhan, Hubei, China.


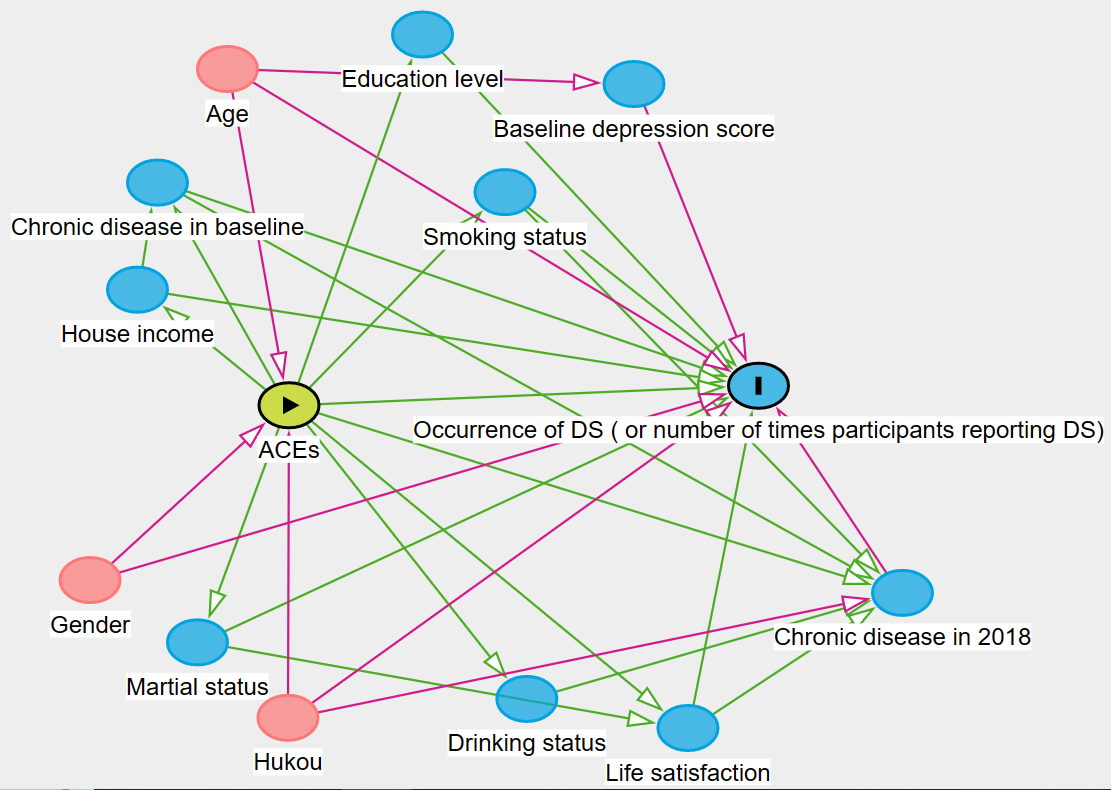


Figure S1 Directed acyclic graph for the association between ACEs and new occurrence of DSs or number of times participants reporting DSs.

The red circle represents the covariate that needs to be adjusted.

| Table S1 Baseline characteristics between included and excluded participants | | | | | |
| --- | --- | --- | --- | --- | --- |
|  | level | Overall  n (%) | Excluded  n (%) | Included  n (%) | *P* value |
| Sample size (n) |  | 17705 | 13726 | 3979 |  |
| Age (mean (SD)) (year) |  | 59.05 (10.15) | 59.80 (10.61) | 56.48 (7.85) | <0.001 |
| Gender | Male | 8471 (47.9) | 6352 (46.3) | 2119 (53.3) | <0.001 |
|  | Female | 9221 (52.1) | 7365 (53.7) | 1856 (46.7) |  |
| Education | Elementary school or below | 11755 (66.6) | 9547 (69.8) | 2208 (55.5) | <0.001 |
|  | Middle/high/vocational school | 5469 (31.0) | 3795 (27.8) | 1674 (42.1) |  |
|  | Equal or more than associate degree | 429 (2.4) | 333 (2.4) | 96 (2.4) |  |
| Marital status | Married | 14170 (80.2) | 10594 (77.4) | 3576 (89.9) | <0.001 |
|  | Separated | 1330 (7.5) | 1182(8.6) | 148 (3.8) |  |
|  | Never-Married/Divorced /Widowed/ | 2175 (12.3) | 1920(14.0) | 255(6.4) |  |
| Hukou | Agricultural | 13694 (77.5) | 10617 (77.5) | 3077 (77.4) | 0.042 |
|  | Non-agricultural | 3862 (21.9) | 2995 (21.9) | 867 (21.8) |  |
|  | Unified Residence | 107 (0.6) | 73 (0.5) | 34 (0.9) |  |
| Chronic disease | No | 5603 (31.6) | 4045 (29.5) | 1558 (39.2) | <0.001 |
|  | Yes | 11807 (66.7) | 9429 (68.7) | 2378 (59.8) |  |
|  | missing | 295 (1.7) | 252 (1.8) | 43 (1.1) |  |
| Smoke status (%) | Non-smoker | 11394 (64.4) | 9078 (66.1) | 2316 (58.2) | <0.001 |
|  | Ex-smoker | 1440 (8.1) | 1108 (8.1) | 332 (8.3) |  |
|  | Smoker | 4871 (27.5) | 3540 (25.8) | 1331 (33.5) |  |
| Drinking status (%) | ≥ 1 time/month | 4383 (25.0) | 3219 (23.7) | 1164 (29.3) | <0.001 |
|  | < 1 time/month | 1384 (7.9) | 1045 (7.7) | 339 (8.5) |  |
|  | Non-drinker | 11783 (67.1) | 9307 (68.6) | 2476 (62.2) |  |
| Life satisfaction | Satisfied | 3144 (21.8) | 2138 (20.1) | 1006 (27.0) | <0.001 |
|  | Somewhat satisfied | 8941 (62.3) | 6483 (61.0) | 2458 (65.9) |  |
|  | Dissatisfied | 2276 (15.8) | 2012 (18.9) | 264 (7.1) |  |
| House income (yuan/year) | < 27600 | 9524 (54.9) | 7541 (56.3) | 1983 (50.0) | <0.001 |
|  | ≥27600 | 7831 (45.1) | 5847 (43.7) | 1984 (50.0) |  |

| Table S2 Associations between ACEs and new occurrence of DSs in Cox proportional hazards model | | | | | | |
| --- | --- | --- | --- | --- | --- | --- |
|  | Unadjusted |  | Adjusted model 1 |  | Adjusted model 2 |  |
| Total ACE | HR | *P* value | HR | *P* value | HR | *P* value |
| 0 | ref |  | ref |  | ref |  |
| 1 | 1.159 (0.949, 1.415) | 0.149 | 1.008[0.790, 1.285] | 0.95 | 0.991 [0.777, 1.264] | 0.944 |
| 2 | 1.161 (0.951, 1.416) | 0.143 | 1.037[0.814, 1.321] | 0.77 | 0.996 [0.781, 1.269] | 0.972 |
| 3+ | 1.712 (1.424, 2.058) | <0.001 | 1.589[1.271, 1.986] | <0.001 | 1.515 [1.212, 1.894] | <0.001 |
| *P* for trend | <0.001 |  | <0.001 |  | <0.001 |  |
| Deprivation-related ACE |  |  |  |  |  |  |
|  | ref |  | ref |  | ref |  |
| 1 | 1.140 (0.977, 1.330) | 0.095 | 1.075 [0.888, 1.303] | 0.457 | 1.069 [0.883, 1.296] | 0.493 |
| 2 | 1.188 (1.017, 1.389) | 0.03 | 1.104 [0.908, 1.342] | 0.319 | 1.080 [0.888, 1.313] | 0.439 |
| 3+ | 1.809 (1.535, 2.131) | <0.001 | 1.562 [1.270, 1.920] | <0.001 | 1.488 [1.210, 1.831] | <0.001 |
| *P* for trend | <0.001 |  | <0.001 |  | <0.001 |  |
| Threat-related ACE |  |  |  |  |  |  |
| 0 | ref |  | ref |  | ref |  |
| 1 | 1.272 (1.127, 1.436) | <0.001 | 1.301 [1.123, 1.507] | <0.001 | 1.274 [1.099, 1.476] | 0.001 |
| 2 | 1.342 (1.160, 1.553) | <0.001 | 1.494 [1.246, 1.791] | <0.001 | 1.467 [1.223, 1.759] | <0.001 |
| 3+ | 1.688 (1.461, 1.950) | <0.001 | 1.632 [1.361, 1.957] | <0.001 | 1.570 [1.308, 1.884] | <0.001 |
| *P* for trend | <0.001 |  | <0.001 |  | <0.001 |  |
| LCA driven ACE |  |  |  |  |  |  |
| Low risk | ref |  | ref |  | ref |  |
| Poor parent relationship | 1.788 [1.498, 2.134] | <0.001 | 1.644 [1.319, 2.051] | <0.001 | 1.607 [1.289, 2.004] | <0.001 |
| Physical abuse | 1.298 [1.142, 1.475] | <0.001 | 1.486 [1.267, 1.742] | <0.001 | 1.426 [1.216, 1.671] | <0.001 |
| Biological parent absence | 1.207 [0.854, 1.707] | 0.287 | 1.114 [0.734, 1.691] | 0.612 | 1.166 [0.767, 1.774] | 0.472 |
| Mental neglect | 1.146 [0.951, 1.381] | 0.153 | 1.179 [0.939, 1.480] | 0.156 | 1.184 [0.943, 1.485] | 0.146 |

Adjusted model 1: Adjusted for deprivation-related ACEs (if applicable), threat-related ACEs (if applicable), age, gender, marital status, education level,

Hukou, chronic disease, smoking status, drinking status, life satisfaction, house income, and chronic disease in 2018. Adjusted model 2: Additionally adjusted for CES-D-10 scores at baseline based on model 1.

| Table S3 Associations between ACEs and new occurrence of DSs according to gender in the Cox proportional hazards model | | | | | | |
| --- | --- | --- | --- | --- | --- | --- |
|  | Unadjusted |  | Adjusted model 1 |  | Adjusted model 2 |  |
| Male |  |  |  |  |  |  |
| Total ACE | HR | *P* value | HR | *P* value | HR | *P* value |
| 0 | ref |  | ref |  | ref |  |
| 1 | 1.156 [0.838, 1.593] | 0.377 | 0.972 [0.663, 1.426] | 0.885 | 0.970 [0.660, 1.424] | 0.876 |
| 2 | 1.020 [0.740, 1.406] | 0.904 | 0.888 [0.605, 1.302] | 0.543 | 0.860 [0.586, 1.262] | 0.440 |
| 3+ | 1.655 [1.234, 2.221] | 0.001 | 1.419 [1.004, 2.006] | 0.048 | 1.361 [0.962, 1.925] | 0.082 |
| *P* for trend | <0.001 |  | <0.001 |  | 0.001 |  |
| Deprivation-related ACE |  |  |  |  |  |  |
| 0 | ref |  | ref |  | ref |  |
| 1 | 1.300 [1.014, 1.667] | 0.038 | 1.130 [0.827, 1.543] | 0.443 | 1.117 [0.818, 1.526] | 0.486 |
| 2 | 1.220 [0.948, 1.569] | 0.122 | 1.043 [0.761, 1.429] | 0.794 | 0.989 [0.721, 1.356] | 0.944 |
| 3+ | 1.947 [1.499, 2.530] | <0.001 | 1.586 [1.137, 2.214] | 0.007 | 1.488 [1.065, 2.079] | 0.020 |
| *P* for trend | <0.001 |  | 0.006 |  | 0.030 |  |
| Threat-related ACE |  |  |  |  |  |  |
| 0 | ref |  | ref |  | ref |  |
| 1 | 1.223 [1.016, 1.474] | 0.034 | 1.320 [1.056, 1.649] | 0.015 | 1.188 [0.984, 1.434] | 0.073 |
| 2 | 1.383 [1.128, 1.696] | 0.002 | 1.470 [1.140, 1.894] | 0.003 | 1.320 [1.073, 1.624] | 0.009 |
| 3+ | 1.664 [1.355, 2.043] | <0.001 | 1.519 [1.176, 1.962] | 0.001 | 1.508 [1.221, 1.863] | <0.001 |
| *P* for trend | <0.001 |  | <0.001 |  | <0.001 |  |
| LCA driven ACE | |  |  |  |  |  |
| Low risk | ref |  | ref |  | ref |  |
| Poor parent relationship | 1.745 [1.332, 2.286] | <0.001 | 1.496 [1.068, 2.096] | 0.019 | 1.510 [1.078, 2.117] | 0.017 |
| Physical abuse | 1.438 [1.212, 1.708] | <0.001 | 1.441 [1.168, 1.778] | 0.001 | 1.409 [1.141, 1.740] | 0.001 |
| Biological parent absence | 1.345 [0.804, 2.251] | 0.259 | 1.445 [0.807, 2.590] | 0.216 | 1.510 [0.843, 2.706] | 0.166 |
| Mental neglect | 1.192 [0.896, 1.585] | 0.228 | 1.295 [0.920, 1.823] | 0.138 | 1.274 [0.905, 1.795] | 0.165 |
| Female |  |  |  |  |  |  |
| Total ACE | HR | *P* value | HR | *P* value | HR | *P* value |
| 0 | ref |  | ref |  | ref |  |
| 1 | 1.190 [0.921, 1.537] | 0.183 | 0.992 [0.723, 1.361] | 0.959 | 0.944 [0.688, 1.296] | 0.723 |
| 2 | 1.345 [1.042, 1.734] | 0.023 | 1.125 [0.821, 1.540] | 0.464 | 1.073 [0.783, 1.470] | 0.660 |
| 3+ | 1.994 [1.572, 2.529] | <0.001 | 1.715 [1.279, 2.300] | <0.001 | 1.631 [1.216, 2.188] | 0.001 |
| *P* for trend | <0.001 |  | <0.001 |  | <0.001 |  |
| Deprivation-related ACE |  |  |  |  |  |  |
| 0 | ref |  | ref |  | ref |  |
| 1 | 1.105 [0.905, 1.349] | 0.325 | 1.031 [0.804, 1.323] | 0.808 | 1.016 [0.793, 1.303] | 0.899 |
| 2 | 1.297 [1.061, 1.584] | 0.011 | 1.164 [0.903, 1.500] | 0.242 | 1.166 [0.905, 1.503] | 0.235 |
| 3+ | 1.875 [1.514, 2.321] | <0.001 | 1.598 [1.219, 2.096] | 0.001 | 1.537 [1.172, 2.017] | 0.002 |
| *P* for trend | <0.001 |  | <0.001 |  | 0.001 |  |
| Threat-related ACE |  |  |  |  |  |  |
| 0 | ref |  | ref |  | ref |  |
| 1 | 1.355 [1.155, 1.590] | <0.001 | 1.281 [1.050, 1.562] | 0.015 | 1.254 [1.028, 1.529] | 0.026 |
| 2 | 1.489 [1.203, 1.842] | <0.001 | 1.492 [1.143, 1.948] | 0.003 | 1.442 [1.104, 1.883] | 0.007 |
| 3+ | 1.991 [1.622, 2.445] | <0.001 | 1.776 [1.368, 2.304] | <0.001 | 1.709 [1.315, 2.221] | <0.001 |
| *P* for trend | <0.001 |  | <0.001 |  | <0.001 |  |
| LCA driven ACE | |  |  |  |  |  |
| Low risk | ref |  | ref |  | ref |  |
| Poor parent relationship | 1.875 [1.481, 2.373] | <0.001 | 1.890 [1.412, 2.529] | <0.001 | 1.807 [1.350, 2.419] | <0.001 |
| Physical abuse | 1.407 [1.152, 1.718] | <0.001 | 1.511 [1.183, 1.931] | 0.001 | 1.452 [1.136, 1.855] | 0.003 |
| Biological parent absence | 1.057 [0.653, 1.711] | 0.822 | 0.865 [0.459, 1.629] | 0.653 | 0.941 [0.499, 1.774] | 0.851 |
| Mental neglect | 1.113 [0.868, 1.427] | 0.398 | 1.140 [0.840, 1.548] | 0.401 | 1.162 [0.855, 1.580] | 0.338 |

Adjusted model 1: Adjusted for deprivation-related ACEs (if applicable), threat-related ACEs (if applicable), age, marital status, education level, Hukou, chronic disease, smoking status, drinking status, life satisfaction, house income, and chronic disease in 2018. Adjusted model 2: Additionally adjusted for CES-D-10 scores at baseline based on model 1.

| Table S4 Associations between ACEs and new occurrence of DSs according to age in Cox proportional hazards model | | | | | | |
| --- | --- | --- | --- | --- | --- | --- |
|  | Unadjusted |  | Adjusted model 1 |  | Adjusted model 2 |  |
| 45~59 years old | |  |  |  |  |  |
| Total ACE | HR | *P* value | HR | *P* value | HR | *P* value |
| 0 | ref |  | ref |  | ref |  |
| 1 | 1.159 [0.920, 1.460] | 0.209 | 1.054 [0.797, 1.394] | 0.713 | 1.022 [0.773, 1.352] | 0.878 |
| 2 | 1.139 [0.904, 1.434] | 0.27 | 1.072 [0.810, 1.418] | 0.628 | 1.009 [0.763, 1.335] | 0.950 |
| 3+ | 1.621 [1.314, 1.999] | <0.001 | 1.656 [1.284, 2.136] | <0.001 | 1.539 [1.192, 1.986] | 0.001 |
| *P* for trend | <0.001 |  | <0.001 |  | <0.001 |  |
| Deprivation-related ACE |  |  |  |  |  |  |
| 0 | ref |  | ref |  | ref |  |
| 1 | 1.175 [0.983, 1.406] | 0.077 | 1.114 [0.929, 1.336] | 0.245 | 1.064 [0.887, 1.277] | 0.506 |
| 2 | 1.199 [1.000, 1.438] | 0.050 | 1.127 [0.937, 1.355] | 0.205 | 1.076 [0.894, 1.295] | 0.438 |
| 3+ | 1.840 [1.517, 2.232] | <0.001 | 1.533 [1.256, 1.872] | <0.001 | 1.444 [1.182, 1.765] | <0.001 |
| *P* for trend | <0.001 |  | <0.001 |  | 0.001 |  |
| Threat-related ACE |  |  |  |  |  |  |
| 0 | ref |  | ref |  | ref |  |
| 1 | 1.201 [1.035, 1.394] | 0.016 | 1.214 [1.016, 1.452] | 0.033 | 1.160 [0.970, 1.386] | 0.104 |
| 2 | 1.294 [1.088, 1.539] | 0.004 | 1.585 [1.284, 1.958] | <0.001 | 1.510 [1.223, 1.865] | <0.001 |
| 3+ | 1.573 [1.324, 1.867] | <0.001 | 1.628 [1.319, 2.010] | <0.001 | 1.552 [1.257, 1.918] | <0.001 |
| *P* for trend | <0.001 |  | <0.001 |  | <0.001 |  |
| LCA driven ACE | |  |  |  |  |  |
| Low risk | ref |  | ref |  | ref |  |
| Poor parent relationship | 1.627 [1.316, 2.012] | <0.001 | 1.718 [1.335, 2.212] | <0.001 | 1.648 [1.280, 2.122] | <0.001 |
| Physical abuse | 1.261 [1.085, 1.466] | 0.002 | 1.548 [1.291, 1.855] | <0.001 | 1.472 [1.228, 1.765] | <0.001 |
| Biological parent absence | 0.879 [0.527, 1.466] | 0.621 | 0.770 [0.423, 1.405] | 0.395 | 0.814 [0.445, 1.490] | 0.505 |
| Mental neglect | 1.104 [0.874, 1.394] | 0.409 | 1.136 [0.863, 1.495] | 0.363 | 1.126 [0.856, 1.480] | 0.397 |
| ≥60 years old | |  |  |  |  |  |
| Total ACE | HR | *P* value | HR | *P* value | HR | *P* value |
| 0 | ref |  | ref |  | ref |  |
| 1 | 1.253 [0.829, 1.892] | 0.284 | 0.967 [0.585, 1.598] | 0.895 | 0.966 [0.584, 1.597] | 0.892 |
| 2 | 1.306 [0.867, 1.966] | 0.201 | 0.964 [0.587, 1.582] | 0.884 | 0.962 [0.586, 1.579] | 0.877 |
| 3+ | 2.053 [1.390, 3.031] | <0.001 | 1.552 [0.967, 2.490] | 0.069 | 1.544 [0.961, 2.481] | 0.072 |
| *P* for trend | <0.001 |  | <0.001 |  | <0.001 |  |
| Deprivation-related ACE |  |  |  |  |  |  |
| 0 | ref |  | ref |  | ref |  |
| 1 | 1.106 [0.810, 1.509] | 0.527 | 1.089 [0.793, 1.496] | 0.598 | 1.081 [0.787, 1.485] | 0.630 |
| 2 | 1.200 [0.878, 1.640] | 0.253 | 1.123 [0.815, 1.546] | 0.478 | 1.118 [0.812, 1.539] | 0.496 |
| 3+ | 1.807 [1.309, 2.495] | <0.001 | 1.559 [1.120, 2.171] | 0.008 | 1.556 [1.116, 2.168] | 0.009 |
| P for trend | <0.001 |  | 0.002 |  | 0.003 |  |
| Threat-related ACE |  |  |  |  |  |  |
| 0 | ref |  | ref |  | ref |  |
| 1 | 1.415 [1.150, 1.741] | 0.001 | 1.539 [1.178, 2.012] | 0.002 | 1.533 [1.173, 2.003] | 0.002 |
| 2 | 1.423 [1.086, 1.866] | 0.011 | 1.291 [0.888, 1.878] | 0.181 | 1.318 [0.906, 1.918] | 0.149 |
| 3+ | 1.962 [1.501, 2.565] | <0.001 | 1.779 [1.225, 2.582] | 0.002 | 1.805 [1.245, 2.618] | 0.002 |
| P for trend | <0.001 |  | <0.001 |  | <0.001 |  |
| LCA driven ACE | |  |  |  |  |  |
| Low risk | |  | ref |  | ref |  |
| Poor parent relationship | 2.215 [1.608, 3.051] | <0.001 | 1.867 [1.334, 2.615] | <0.001 | 1.870 [1.336, 2.619] | <0.001 |
| Physical abuse | 1.355 [1.059, 1.734] | 0.016 | 1.482 [1.150, 1.912] | 0.002 | 1.458 [1.131, 1.880] | 0.004 |
| Biological parent absence | 1.773 [1.104, 2.847] | 0.018 | 1.410 [0.871, 2.282] | 0.162 | 1.396 [0.862, 2.261] | 0.175 |
| Mental neglect | 1.231 [0.903, 1.679] | 0.189 | 1.229 [0.894, 1.690] | 0.204 | 1.230 [0.894, 1.692] | 0.203 |

Adjusted model 1: Adjusted for deprivation-related ACEs (if applicable), threat-related ACEs (if applicable), gender, marital status, education level,

Hukou, chronic disease, smoking status, drinking status, life satisfaction, house income, and chronic disease in 2018. Adjusted model 2: Additionally adjusted for CES-D-10 scores at baseline based on model 1.

| Table S5 Associations between ACEs and new occurrence of DSs according in the Cox proportional hazards model | | | | | | |
| --- | --- | --- | --- | --- | --- | --- |
|  | Unadjusted |  | Adjusted model 1 |  | Adjusted model 2 |  |
| Total ACE | HR | *P* value | HR | *P* value | HR | *P* value |
| 0 | ref |  | ref |  | ref |  |
| 1 | 1.159 (0.949, 1.415) | 0.149 | 1.092 [0.893, 1.335] | 0.392 | 1.062 [0.868, 1.299] | 0.560 |
| 2 | 1.161 (0.951, 1.416) | 0.143 | 1.101 [0.900, 1.346] | 0.349 | 1.066 [0.871, 1.303] | 0.536 |
| 3+ | 1.712 (1.424, 2.058) | <0.001 | 1.662 [1.380, 2.002] | <0.001 | 1.609 [1.336, 1.939] | <0.001 |
| *P* for trend | <0.001 |  | <0.001 |  | <0.001 |  |
| Deprivation-related ACE |  |  |  |  |  |  |
| 0 | ref |  | ref |  | ref |  |
| 1 | 1.140 (0.977, 1.330) | 0.095 | 1.096 [0.938, 1.281] | 0.249 | 1.060 [0.906, 1.239] | 0.467 |
| 2 | 1.188 (1.017, 1.389) | 0.03 | 1.112 [0.949, 1.302] | 0.190 | 1.072 [0.915, 1.257] | 0.387 |
| 3+ | 1.809 (1.535, 2.131) | <0.001 | 1.571 [1.327, 1.858] | <0.001 | 1.481 [1.251, 1.753] | <0.001 |
| *P* for trend | <0.001 |  | <0.001 |  | <0.001 |  |
| Threat-related ACE |  |  |  |  |  |  |
| 0 | ref |  | ref |  | ref |  |
| 1 | 1.272 (1.127, 1.436) | <0.001 | 1.282 [1.134, 1.448] | <0.001 | 1.256 [1.112, 1.420] | <0.001 |
| 2 | 1.342 (1.160, 1.553) | <0.001 | 1.458 [1.256, 1.692] | <0.001 | 1.411 [1.216, 1.638] | <0.001 |
| 3+ | 1.688 (1.461, 1.950) | <0.001 | 1.693 [1.460, 1.963] | <0.001 | 1.613 [1.391, 1.871] | <0.001 |
| *P* for trend | <0.001 |  | <0.001 |  | <0.001 |  |
| LCA driven ACE |  |  |  |  |  |  |
| Low risk | ref |  | ref |  | ref |  |
| Poor parent relationship | 1.788 [1.498, 2.134] | <0.001 | 1.830 [1.532, 2.185] | <0.001 | 1.750 [1.465, 2.090] | <0.001 |
| Physical abuse | 1.298 [1.142, 1.475] | <0.001 | 1.453 [1.275, 1.655] | <0.001 | 1.381 [1.212, 1.574] | <0.001 |
| Biological parent absence | 1.207 [0.854, 1.707] | 0.287 | 1.143 [0.808, 1.617] | 0.451 | 1.159 [0.820, 1.640] | 0.403 |
| Mental neglect | 1.146 [0.951, 1.381] | 0.153 | 1.149 [0.952, 1.386] | 0.148 | 1.140 [0.944, 1.375] | 0.173 |

Adjusted model 1: Adjusted for deprivation-related ACEs (if applicable), threat-related ACEs (if applicable), age, gender, marital status, education level,

Hukou, chronic disease, smoking status, drinking status, and house income. Adjusted model 2: Additionally adjusted for CES-D-10 scores at baseline based on model 1.

| Table S6 Associations between ACEs and new occurrence of DSs according to gender in the Cox proportional hazards model | | | | | | |
| --- | --- | --- | --- | --- | --- | --- |
|  | Unadjusted |  | Adjusted model 1 |  | Adjusted model 2 |  |
| Male |  |  |  |  |  |  |
| Total ACE | HR | *P* value | HR | *P* value | HR | *P* value |
| 0 | ref |  | ref |  | ref |  |
| 1 | 1.156 [0.838, 1.593] | 0.377 | 1.122 [0.812, 1.549] | 0.485 | 1.084 [0.784, 1.498] | 0.625 |
| 2 | 1.020 [0.740, 1.406] | 0.904 | 0.970 [0.702, 1.341] | 0.855 | 0.928 [0.671, 1.282] | 0.650 |
| 3+ | 1.655 [1.234, 2.221] | 0.001 | 1.580 [1.176, 2.123] | 0.002 | 1.460 [1.086, 1.963] | 0.012 |
| *P* for trend | <0.001 |  | <0.001 |  | <0.001 |  |
| Deprivation-related ACE |  |  |  |  |  |  |
| 0 | ref |  | ref |  | ref |  |
| 1 | 1.300 [1.014, 1.667] | 0.038 | 1.188 [0.924, 1.528] | 0.179 | 1.131 [0.879, 1.456] | 0.337 |
| 2 | 1.220 [0.948, 1.569] | 0.122 | 1.082 [0.838, 1.397] | 0.545 | 1.011 [0.783, 1.307] | 0.931 |
| 3+ | 1.947 [1.499, 2.530] | <0.001 | 1.627 [1.245, 2.127] | <0.001 | 1.489 [1.138, 1.947] | 0.004 |
| *P* for trend | <0.001 |  | 0.001 |  | 0.010 |  |
| Threat-related ACE |  |  |  |  |  |  |
| 0 | ref |  | ref |  | ref |  |
| 1 | 1.223 [1.016, 1.474] | 0.034 | 1.197 [0.992, 1.444] | 0.061 | 1.189 [0.986, 1.435] | 0.071 |
| 2 | 1.383 [1.128, 1.696] | 0.002 | 1.368 [1.113, 1.682] | 0.003 | 1.352 [1.099, 1.662] | 0.004 |
| 3+ | 1.664 [1.355, 2.043] | <0.001 | 1.635 [1.327, 2.014] | <0.001 | 1.599 [1.297, 1.970] | <0.001 |
| *P* for trend | <0.001 |  | <0.001 |  | <0.001 |  |
| LCA driven ACE |  |  |  |  |  |  |
| Low risk | ref |  | ref |  | ref |  |
| Poor parent relationship | 1.745 [1.332, 2.286] | <0.001 | 1.760 [1.342, 2.308] | <0.001 | 1.754 [1.337, 2.302] | <0.001 |
| Physical abuse | 1.438 [1.212, 1.708] | <0.001 | 1.473 [1.239, 1.750] | <0.001 | 1.412 [1.188, 1.679] | <0.001 |
| Biological parent absence | 1.345 [0.804, 2.251] | 0.259 | 1.213 [0.723, 2.033] | 0.464 | 1.289 [0.769, 2.161] | 0.335 |
| Mental neglect | 1.192 [0.896, 1.585] | 0.228 | 1.182 [0.888, 1.572] | 0.252 | 1.140 [0.857, 1.517] | 0.368 |
| Female |  |  |  |  |  |  |
| Total ACE | HR | *P* value | HR | *P* value | HR | *P* value |
| 0 | ref |  | ref |  | ref |  |
| 1 | 1.190 [0.921, 1.537] | 0.183 | 1.083 [0.837, 1.403] | 0.544 | 1.022 [0.789, 1.325] | 0.867 |
| 2 | 1.345 [1.042, 1.734] | 0.023 | 1.220 [0.943, 1.577] | 0.13 | 1.148 [0.887, 1.485] | 0.295 |
| 3+ | 1.994 [1.572, 2.529] | <0.001 | 1.845 [1.453, 2.344] | <0.001 | 1.727 [1.359, 2.195] | <0.001 |
| *P* for trend | <0.001 |  | <0.001 |  | <0.001 |  |
| Deprivation-related ACE |  |  |  |  |  |  |
| 0 | ref |  | ref |  | ref |  |
| 1 | 1.105 [0.905, 1.349] | 0.325 | 1.026 [0.838, 1.256] | 0.803 | 0.996 [0.814, 1.220] | 0.971 |
| 2 | 1.297 [1.061, 1.584] | 0.011 | 1.148 [0.937, 1.407] | 0.183 | 1.131 [0.922, 1.386] | 0.237 |
| 3+ | 1.875 [1.514, 2.321] | <0.001 | 1.559 [1.252, 1.943] | <0.001 | 1.501 [1.204, 1.871] | <0.001 |
| *P* for trend | <0.001 |  | <0.001 |  | <0.001 |  |
| Threat-related ACE |  |  |  |  |  |  |
| 0 | ref |  | ref |  | ref |  |
| 1 | 1.355 [1.155, 1.590] | <0.001 | 1.363 [1.158, 1.603] | <0.001 | 1.329 [1.129, 1.564] | 0.001 |
| 2 | 1.489 [1.203, 1.842] | <0.001 | 1.553 [1.247, 1.934] | <0.001 | 1.487 [1.195, 1.852] | <0.001 |
| 3+ | 1.991 [1.622, 2.445] | <0.001 | 1.726 [1.396, 2.134] | <0.001 | 1.617 [1.306, 2.001] | <0.001 |
| *P* for trend | <0.001 |  | <0.001 |  | <0.001 |  |
| LCA driven ACE |  |  |  |  |  |  |
| Low risk | ref |  | ref |  | ref |  |
| Poor parent relationship | 1.875 [1.481, 2.373] | <0.001 | 1.896 [1.497, 2.401] | <0.001 | 1.768 [1.395, 2.240] | <0.001 |
| Physical abuse | 1.407 [1.152, 1.718] | <0.001 | 1.408 [1.151, 1.723] | 0.001 | 1.327 [1.084, 1.624] | 0.006 |
| Biological parent absence | 1.057 [0.653, 1.711] | 0.822 | 1.043 [0.644, 1.690] | 0.864 | 1.043 [0.644, 1.690] | 0.864 |
| Mental neglect | 1.113 [0.868, 1.427] | 0.398 | 1.128 [0.877, 1.450] | 0.349 | 1.133 [0.881, 1.457] | 0.329 |

Adjusted model 1: Adjusted for deprivation-related ACEs (if applicable), threat-related ACEs (if applicable), age, marital status, education level, Hukou, chronic disease, smoking status, drinking status, and house income. Adjusted model 2: Additionally adjusted for CES-D-10 scores at baseline based on model 1.

| Table S7 Associations between ACEs and new occurrence of DSs according to age in Cox proportional hazards model | | | | | | |
| --- | --- | --- | --- | --- | --- | --- |
|  | Unadjusted |  | Adjusted model 1 |  | Adjusted model 2 |  |
| 45~59 years old |  |  |  |  |  |  |
| Total ACE | HR | *P* value | HR | *P* value | HR | *P* value |
| 0 | ref |  | ref |  | ref |  |
| 1 | 1.159 [0.920, 1.460] | 0.209 | 1.120 [0.888, 1.411] | 0.339 | 1.083 [0.859, 1.366] | 0.498 |
| 2 | 1.139 [0.904, 1.434] | 0.27 | 1.111 [0.881, 1.401] | 0.375 | 1.053 [0.835, 1.329] | 0.662 |
| 3+ | 1.621 [1.314, 1.999] | <0.001 | 1.664 [1.346, 2.056] | <0.001 | 1.537 [1.243, 1.901] | <0.001 |
| *P* for trend | <0.001 |  | <0.001 |  | <0.001 |  |
| Deprivation-related ACE |  |  |  |  |  |  |
| 0 | ref |  | ref |  | ref |  |
| 1 | 1.175 [0.983, 1.406] | 0.077 | 1.127 [0.941, 1.351] | 0.194 | 1.076 [0.897, 1.291] | 0.428 |
| 2 | 1.199 [1.000, 1.438] | 0.05 | 1.131 [0.941, 1.360] | 0.191 | 1.084 [0.901, 1.304] | 0.394 |
| 3+ | 1.840 [1.517, 2.232] | <0.001 | 1.583 [1.298, 1.931] | <0.001 | 1.473 [1.207, 1.798] | <0.001 |
| *P* for trend | <0.001 |  | <0.001 |  | <0.001 |  |
| Threat-related ACE |  |  |  |  |  |  |
| 0 | ref |  | ref |  | ref |  |
| 1 | 1.201 [1.035, 1.394] | 0.016 | 1.211 [1.042, 1.408] | 0.013 | 1.170 [1.006, 1.361] | 0.041 |
| 2 | 1.294 [1.088, 1.539] | 0.004 | 1.455 [1.218, 1.738] | <0.001 | 1.408 [1.180, 1.681] | <0.001 |
| 3+ | 1.573 [1.324, 1.867] | <0.001 | 1.596 [1.337, 1.904] | <0.001 | 1.511 [1.265, 1.804] | <0.001 |
| *P* for trend | <0.001 |  | <0.001 |  | <0.001 |  |
| LCA driven ACE |  |  |  |  |  |  |
| Low risk | ref |  | ref |  | ref |  |
| Poor parent relationship | 1.627 [1.316, 2.012] | <0.001 | 1.730 [1.398, 2.140] | <0.001 | 1.634 [1.320, 2.022] | <0.001 |
| Physical abuse | 1.261 [1.085, 1.466] | 0.002 | 1.430 [1.228, 1.667] | <0.001 | 1.347 [1.156, 1.571] | <0.001 |
| Biological parent absence | 0.879 [0.527, 1.466] | 0.621 | 0.853 [0.511, 1.423] | 0.542 | 0.870 [0.521, 1.452] | 0.595 |
| Mental neglect | 1.104 [0.874, 1.394] | 0.409 | 1.121 [0.886, 1.418] | 0.341 | 1.109 [0.877, 1.403] | 0.389 |
| ≥60 years old |  |  |  |  |  |  |
| Total ACE | HR | *P* value | HR | *P* value | HR | *P* value |
| 0 | ref |  | ref |  | ref |  |
| 1 | 1.253 [0.829, 1.892] | 0.284 | 1.188 [0.784, 1.799] | 0.416 | 1.101 [0.727, 1.669] | 0.649 |
| 2 | 1.306 [0.867, 1.966] | 0.201 | 1.252 [0.829, 1.891] | 0.286 | 1.169 [0.774, 1.767] | 0.458 |
| 3+ | 2.053 [1.390, 3.031] | <0.001 | 2.086 [1.408, 3.091] | <0.001 | 1.944 [1.311, 2.882] | <0.001 |
| *P* for trend | <0.001 |  | <0.001 |  | <0.001 |  |
| Deprivation-related ACE |  |  |  |  |  |  |
| 0 | ref |  | ref |  | ref |  |
| 1 | 1.106 [0.810, 1.509] | 0.527 | 1.082 [0.788, 1.486] | 0.625 | 1.081 [0.787, 1.484] | 0.630 |
| 2 | 1.200 [0.878, 1.640] | 0.253 | 1.136 [0.826, 1.564] | 0.432 | 1.124 [0.817, 1.547] | 0.471 |
| 3+ | 1.807 [1.309, 2.495] | <0.001 | 1.603 [1.152, 2.230] | 0.005 | 1.583 [1.138, 2.203] | 0.006 |
| *P* for trend | <0.001 |  | 0.001 |  | 0.002 |  |
| Threat-related ACE |  |  |  |  |  |  |
| 0 | ref |  | ref |  | ref |  |
| 1 | 1.415 [1.150, 1.741] | 0.001 | 1.439 [1.166, 1.777] | 0.001 | 1.446 [1.170, 1.785] | 0.001 |
| 2 | 1.423 [1.086, 1.866] | 0.011 | 1.467 [1.111, 1.938] | 0.007 | 1.411 [1.068, 1.864] | 0.015 |
| 3+ | 1.962 [1.501, 2.565] | <0.001 | 2.069 [1.571, 2.724] | <0.001 | 1.990 [1.511, 2.620] | <0.001 |
| *P* for trend | <0.001 |  | <0.001 |  | <0.001 |  |
| LCA driven ACE |  |  |  |  |  |  |
| Low risk |  |  | ref |  | ref |  |
| Poor parent relationship | 2.215 [1.608, 3.051] | <0.001 | 2.147 [1.557, 2.962] | <0.001 | 2.104 [1.524, 2.903] | <0.001 |
| Physical abuse | 1.355 [1.059, 1.734] | 0.016 | 1.561 [1.212, 2.009] | 0.001 | 1.514 [1.177, 1.948] | 0.001 |
| Biological parent absence | 1.773 [1.104, 2.847] | 0.018 | 1.529 [0.949, 2.463] | 0.081 | 1.530 [0.950, 2.464] | 0.080 |
| Mental neglect | 1.231 [0.903, 1.679] | 0.189 | 1.197 [0.875, 1.638] | 0.262 | 1.203 [0.879, 1.647] | 0.248 |

Adjusted model 1: Adjusted for deprivation-related ACEs (if applicable), threat-related ACEs (if applicable), gender, marital status, education level,

Hukou, chronic disease, smoking status, drinking status, and house income. Adjusted model 2: Additionally adjusted for CES-D-10 scores at baseline based on model 1.

| Table S8 Associations between ACEs and the number of times with DSs in ordered-logistic regressions model. | | | | | | |
| --- | --- | --- | --- | --- | --- | --- |
|  | Unadjusted |  | Adjusted model 1 |  | Adjusted model 2 |  |
| Total ACE | OR | *P* value | OR | *P* value | OR | *P* value |
| 0 | ref |  |  |  |  |  |
| 1 | 1.226[0.965,1.562] | 0.097 | 1.106[0.862, 1.424] | 0.432 | 1.070[0.831, 1.381] | 0.603 |
| 2 | 1.209[0.953,1.539] | 0.121 | 1.056[0.823, 1.359] | 0.673 | 1.008[0.784, 1.301] | 0.949 |
| 3+ | 2.032[1.628,2.548] | <0.001 | 1.857[1.473, 2.533] | 0.000 | 1.753[1.386, 2.225] | <0.001 |
| *P* for trend | <0.001 |  | <0.001 |  | <0.001 |  |
| Deprivation-related ACE |  |  |  |  |  |  |
| 0 | ref |  | ref |  | ref |  |
| 1 | 1.192[0.988,1.441] | 0.067 | 1.090[0.895, 1.331] | 0.393 | 1.069[0.876, 1.307] | 0.513 |
| 2 | 1.292[1.068,1.565] | 0.009 | 1.119[0.916, 1.369] | 0.274 | 1.097[0.896, 1.345] | 0.372 |
| 3+ | 2.276[1.847,2.808] | <0.001 | 1.779[1.442, 2.247] | <0.001 | 1.704[1.363, 2.132] | <0.001 |
| *P* for trend | <0.001 |  | <0.001 |  | <0.001 |  |
| Threat-related ACE |  |  |  |  |  |  |
| 0 | ref |  | ref |  | ref |  |
| 1 | 1.344[1.153,1.566] | <0.001 | 1.360[1.159, 1.596] | <0.001 | 1.342[1.114, 1.575] | <0.001 |
| 2 | 1.409[1.168,1.696] | <0.001 | 1.493[1.227, 1.816] | <0.001 | 1.437[1.178, 1.750] | <0.001 |
| 3+ | 2.030[1.672,2.462] | <0.001 | 1.900[1.550, 2.326] | <0.001 | 1.830[1.489, 2.246] | <0.001 |
| *P* for trend | <0.001 |  | <0.001 |  | <0.001 |  |
| LCA driven ACE |  |  |  |  |  |  |
| Low risk | Ref |  | Ref ^a^ |  | Ref |  |
| Poor parent relationship | 2.207[1.726, 2.817] | <0.001 | 2.009[1.560, 2.580] | <0.001 | 1.960[1.519, 2.523] | <0.001 |
| Physical abuse | 1.380[1.169, 1.627] | <0.001 | 1.507[1.267, 1.792] | <0.001 | 1.430[1.199, 1.702] | <0.001 |
| Biological parent absence | 1.213[0.773, 1.873] | 0.391 | 1.027[0.642, 1.614] | 0.910 | 1.023[0.636, 1.620] | 0.922 |
| Mental neglect | 1.177[0.927, 1.489] | 0.178 | 1.165[0.908, 1.487] | 0.225 | 1.158[0.900, 1.482] | 0.249 |

Adjusted model 1: Adjusted for deprivation-related ACEs (if applicable), threat-related ACEs (if applicable), age, gender, marital status, education level,

Hukou, chronic disease, smoking status, drinking status, life satisfaction, house income, and chronic disease in 2018. Adjusted model 2: Additionally adjusted for CES-D-10 scores at baseline based on model 1.

| Table S9 Associations between ACEs and the number of times with DSs in ordered-logistic regressions model. | | | | | | |
| --- | --- | --- | --- | --- | --- | --- |
|  | Crude model |  | Adjusted model 1 |  | Adjusted model 2 |  |
| Male |  |  |  |  |  |  |
| Total ACE | OR | *P* value | OR | *P* value | OR | *P* value |
| 0 | ref |  | ref |  | ref |  |
| 1 | 1.175[0.811, 1.721] | 0.399 | 1.022[0.633, 1.411] | 0.912 | 0.986[0.593, 1.380] | 0.946 |
| 2 | 1.020[0.705, 1.492] | 0.917 | 0.847[0.459, 1.236] | 0.402 | 0.792[0.399,1.185] | 0.245 |
| 3+ | 1.862[1.327, 2.653] | <0.001 | 1.501[1.142, 1.859] | 0.026 | 1.386[1.024, 1.749] | 0.077 |
| *P* for trend | <0.001 |  | 0.000 |  | 0.001 |  |
| Deprivation-related ACE |  |  |  |  |  |  |
| 0 | ref |  | ref |  | ref |  |
| 1 | 1.324[0.992, 1.779] | 0.060 | 1.099[0.795, 1.403] | 0.542 | 1.039[0.732, 1.346] | 0.806 |
| 2 | 1.276[0.953, 1.722] | 0.106 | 1.012[0.703, 1.320] | 0.942 | 0.938[0.626, 1.250] | 0.686 |
| 3+ | 2.355[1.719, 3.246] | <0.001 | 1.726[1.392, 2.060] | 0.001 | 1.552[1.214, 1.890] | 0.011 |
| *P* for trend | <0.001 |  | 0.003 |  | 0.019 |  |
| Threat-related ACE |  |  |  |  |  |  |
| 0 | ref |  | ref |  | ref |  |
| 1 | 1.242[0.991, 1.554] | 0.058 | 1.182[0.949, 1.416] | 0.160 | 1.176[0.940, 1.412] | 0.179 |
| 2 | 1.459[1.134, 1.872] | 0.003 | 1.376[1.114, 1.638] | 0.017 | 1.347[1.081, 1.613] | 0.028 |
| 3+ | 1.935[1.489, 2.510] | <0.001 | 1.732[1.459, 2.006] | 0.000 | 1.704[1.428, 1.081] | <0.001 |
| *P* for trend | <0.001 |  | <0.001 |  | <0.001 |  |
| LCA driven ACE |  |  |  |  |  |  |
| Low risk |  |  |  |  |  |  |
| Poor parent relationship | 2.131[1.490, 3.332] | <0.001 | 1.881[1.515, 2.247] | 0.001 | 1.923[1.554, 2.293] | 0.001 |
| Physical abuse | 1.561[1.260, 1.931] | <0.001 | 1.493[1.271, 1.715] | 0.000 | 1.429[1.204, 7.293] | 0.002 |
| Biological parent absence | 1.466[0.752, 2.747] | 0.244 | 1.255[0.593, 1.917] | 0.501 | 1.257[0.590, 9.293] | 0.501 |
| Mental neglect | 1.221[0.875, 1.719] | 0.261 | 1.149[0.790, 1.508] | 0.448 | 1.087[0.724, 5.293] | 0.651 |
| Female |  |  |  |  |  |  |
| Total ACE | OR | *P* value | OR | *P* value | OR |  |
| 0 | ref |  | ref |  | ref |  |
| 1 | 1.318[0.963, 1.813] | 0.087 | 1.121[0.792, 1.450] | 0.497 | 1.076[0.743, 1.408] | 0.666 |
| 2 | 1.480[1.082, 2.036] | 0.015 | 1.208[0.878, 1.537] | 0.261 | 1.158[0.826, 1.490] | 0.386 |
| 3+ | 2.615[1.943, 3.542] | <0.001 | 2.179[1.869, 2.490] | <0.001 | 2.076[1.763, 2.390] | <0.001 |
| *P* for trend | <0.001 |  | <0.001 |  | <0.001 |  |
| Deprivation-related ACE |  |  |  |  |  |  |
| 0 | ref |  | ref |  | ref |  |
| 1 | 1.211[0.942, 1.560] | 0.137 | 1.063[0.799, 1.327] | 0.651 | 1.055[0.789, 1.32] | 0.691 |
| 2 | 1.508[1.167, 1.951] | 0.002 | 1.223[0.954, 1.492] | 0.142 | 1.241[0.970, 1.512] | 0.118 |
| 3+ | 2.520[1.894, 3.361] | <0.001 | 1.854[1.552, 2.157] | <0.001 | 1.818[1.514, 2.123] | <0.001 |
| *P* for trend | <0.001 |  | <0.001 |  | <0.001 |  |
| Threat-related ACE |  |  |  |  |  |  |
| 0 | ref |  | ref |  | ref |  |
| 1 | 1.513[1.222, 1.871] | <0.001 | 1.525[1.303, 1.747] | 0.000 | 1.494[1.270, 1.717] | <0.001 |
| 2 | 1.617[1.210, 2.155] | 0.001 | 1.634[1.332, 1.936] | 0.001 | 1.542[1.239, 1.846] | 0.005 |
| 3+ | 2.614[1.942, 3.517] | <0.001 | 2.099[1.790, 2.408] | 0.000 | 1.973[1.661, 2.284] | <0.001 |
| *P* for trend | <0.001 |  | <0.001 |  | <0.001 |  |
| LCA driven ACE |  |  |  |  |  |  |
| Low risk | ref |  | ref |  | ref |  |
| Poor parent relationship | 2.348[1.665, 3.305] | <0.001 | 2.229[1.519, 2.523] | <0.001 | 2.080[1.727, 2.433] | <0.001 |
| Physical abuse | 1.547[1.174, 2.035] | 0.002 | 1.497[1.199, 1.702] | 0.005 | 1.424[1.139, 1.709] | 0.015 |
| Biological parent absence | 0.992[0.522, 1.829] | 0.981 | 0.914[0.636, 1.620] | 0.787 | 0.893[0.235, 1.552] | 0.738 |
| Mental neglect | 1.149[0.824, 1.595] | 0.409 | 1.178[0.900, 1.482] | 0.346 | 1.245[0.900, 1.591] | 0.214 |

Adjusted model 1: Adjusted for deprivation-related ACEs (if applicable), threat-related ACEs (if applicable), age, marital status, education level,

Hukou, chronic disease, smoking status, drinking status, life satisfaction, house income, and chronic disease in 2018. Adjusted model 2: Additionally adjusted for CES-D-10 scores at baseline based on model 1.

| Table S10 Associations between ACEs and the number of times with DSs according to age in ordered-logistic regressions model. | | | | | | | |
| --- | --- | --- | --- | --- | --- | --- | --- |
|  | Crude model |  | Adjusted model 1 |  | | Adjusted model 2 |  |
| 45-59 years old |  |  |  |  | |  |  |
| Total ACE | OR | *P* value | OR | *P* value | | OR | *P* value |
| 0 | ref |  |  |  | |  |  |
| 1 | 1.233[0.934,1.636] | 0.142 | 1.110[0.817, 1.403] | | 0.485 | 1.093[0.797, 1.390] | 0.555 |
| 2 | 1.192[0.903,1.581] | 0.218 | 1.017[0.724, 1.311] | 0.908 | | 0.973[0.675, 1.270] | 0.855 |
| 3+ | 1.922[1.489,2.493] | <0.001 | 1.776[1.505, 2.047] | 0.000 | | 1.661[1.387, 1.936] | <0.001 |
| *P* for trend | <0.001 |  | <0.001 |  | | <0.001 |  |
| Deprivation-related ACE | |  |  |  | |  |  |
| 0 | ref |  | ref |  | | ref |  |
| 1 | 1.224[0.984,1.527] | 0.071 | 1.103[0.872, 1.335] | 0.405 | | 1.067[0.833, 1.302] | 0.586 |
| 2 | 1.303[1.042,1.631] | 0.020 | 1.101[0.865, 1.337] | 0.423 | | 1.073[0.834, 1.312] | 0.562 |
| 3+ | 2.439[1.901,3.136] | <0.001 | 1.896[1.630, 2.162] | 0.000 | | 1.747[1.479, 2.016] | <0.001 |
| *P* for trend | <0.001 |  | <0.001 |  | | <0.001 |  |
| Threat-related ACE | |  |  |  | |  |  |
| 0 | ref |  | ref |  | | ref |  |
| 1 | 1.253[1.037,1.512] | 0.019 | 1.247[1.050, 1.444] | 0.028 | | 1.214[1.015, 1412] | 0.056 |
| 2 | 1.380[1.104,1.723] | 0.005 | 1.565[1.331, 1.799] | 0.000 | | 1.486[1.250, 1.723] | 0.001 |
| 3+ | 1.851[1.472,2.326] | <0.001 | 1.763[1.520, 2.005] | 0.000 | | 1.669[1.424, 1.915] | <0.001 |
| *P* for trend | <0.001 |  | 0.000 |  | | <0.001 |  |
| LCA driven ACE | |  |  |  | |  |  |
| Low risk | ref |  | ref |  | | ref |  |
| Poor parent relationship | 2.003[1.495, 2.678] | <0.001 | 1.992[1.519, 2.523] | <0.001 | | 1.886[1.584, 2.187] | <0.001 |
| Physical abuse | 1.356[1.116, 1.647] | 0.002 | 1.495[1.199, 1.702] | <0.001 | | 1.402[1.194, 1.610] | 0.001 |
| Biological parent absent | 0.777[0.408, 1.410] | 0.422 | 0.670[0.636, 1.620] | 0.226 | | 0.676[0.016, 1.337] | 0.246 |
| Mental neglect | 1.154[0.853, 1.551] | 0.348 | 1.132[0.900, 1.480] | 0.437 | | 1.124[0.808, 1.439] | 0.469 |
| ≥60 years old |  |  |  |  | |  |  |
| Total ACE | OR | *P* value | OR | *P* value | | OR | *P* value |
| 0 | ref |  | ref |  | | ref |  |
| 1 | 1.323[0.824,2.175] | 0.256 | 1.209[0.708, 1.501] | 0.457 | | 1.111[0.607, 1.615] | 0.683 |
| 2 | 1.362[0.851,2.230] | 0.208 | 1.226[0.726, 1.500] | 0.424 | | 1.145[0.643, 1.648] | 0.597 |
| 3+ | 2.461[1.571,3.959] | <0.001 | 2.287[1.807, 1.480] | 0.001 | | 2.142[1.661, 2.623] | 0.002 |
| *P* for trend | <0.001 |  | <0.001 |  | | <0.001 |  |
| Deprivation-related ACE | |  |  |  | |  |  |
| 0 | ref |  | ref |  | | ref |  |
| 1 | 1.175[0.811, 1.720] | 0.400 | 1.171[0.775, 1.567] | 0.435 | | 1.148[0.749, 1.547] | 0.497 |
| 2 | 1.320[0.909, 1.937] | 0.150 | 1.250[0.839, 1.651] | 0.275 | | 1.237[0.834, 1.640] | 0.301 |
| 3+ | 2.122[1.428, 3.182] | <0.001 | 1.809[1.384, 2.235] | 0.006 | | 1.744[1.315, 2.172] | 0.011 |
| *P* for trend | <0.001 |  | 0.003 |  | | 0.005 |  |
| Threat-related ACE |  |  |  |  | |  |  |
| 0 | ref |  |  |  | |  |  |
| 1 | 1.531[1.176,1.989] | 0.001 | 1.563[1.287, 1.839] | 0.002 | | 1.571[1.293, 1.848] | 0.001 |
| 2 | 1.427[1.010,2.004] | 0.042 | 1.326[0.959, 1.692] | 0.132 | | 1.322[0.951, 1.692] | 0.141 |
| 3+ | 2.479[1.718,3.570] | <0.001 | 2.382[2.000, 2.766] | 0.000 | | 2.415[2.033, 2.797] | <0.001 |
| *P* for trend | <0.001 |  | <0.001 |  | | <0.001 |  |
| LCA driven ACE | |  |  |  | |  |  |
| Low risk | ref |  | ref |  | | ref |  |
| Poor parent relationship | 2.733[1.734, 4.290] | <0.001 | 2.171[1.702, 2.639] | 0.001 | | 1.923[1.554, 2.293] | 0.001 |
| Physical abuse | 1.388[1.009, 1.898] | 0.042 | 1.591[1.258, 1.923] | 0.006 | | 1.429[1.204, 1.653] | 0.002 |
| Biological parent absent | 2.167[1.118, 4.125] | 0.020 | 1.670[0.990, 2.351] | 0.140 | | 1.257[0.590, 1.924] | 0.501 |
| Mental neglect | 1.231[0.827, 1.811] | 0.297 | 1.217[0.809, 1.625] | 0.346 | | 1.087[0.724, 1.451] | 0.651 |

Adjusted model 1: Adjusted for deprivation-related ACEs (if applicable), threat-related ACEs (if applicable), gender, marital status, education level,

Hukou, chronic disease, smoking status, drinking status, life satisfaction, house income, and chronic disease in 2018. Adjusted model 2: Additionally adjusted for CES-D-10 scores at baseline based on model 1.

| Table S11 Associations between ACEs and the number of times with DSs in ordered-logistic regressions model. | | | | | | |
| --- | --- | --- | --- | --- | --- | --- |
|  | Unadjusted |  | Adjusted model 1 |  | Adjusted model 2 |  |
| Total ACE | OR | *P* value | OR | *P* value | OR | *P* value |
| 0 | ref |  | ref |  | ref |  |
| 1 | 1.226[0.965,1.562] | 0.097 | 1.170[0.915, 1.501] | 0.214 | 1.008[0.915, 1.501] | 0.969 |
| 2 | 1.209[0.953,1.539] | 0.121 | 1.149[[0.899, 1.473] | 0.271 | 0.847[0.899, 1.473] | 0.400 |
| 3+ | 2.032[1.628,2.548] | <0.001 | 2.067[1.645, 2.609] | 0.000 | 1.497[1.645, 2.609] | 0.027 |
| *P* for trend | <0.001 |  | <0.001 |  | <0.001 |  |
| Deprivation-related ACE |  |  |  |  |  |  |
| 0 | ref |  | ref |  | ref |  |
| 1 | 1.192[0.988,1.441] | 0.067 | 1.144[0.941, 1.392] | 0.179 | 1.108[0.910, 1.352] | 0.309 |
| 2 | 1.292[1.068,1.565] | 0.009 | 1.190[0.976, 1.453] | 0.086 | 1.158[0.938, 1.428] | 0.152 |
| 3+ | 2.276[1.847,2.808] | <0.001 | 1.931[1.552, 2.405] | <0.001 | 1.796[1.440, 2.243] | <0.001 |
| *P* for trend | <0.001 |  | <0.001 |  | <0.001 |  |
| Threat-related ACE |  |  |  |  |  |  |
| 0 | ref |  | ref |  | ref |  |
| 1 | 1.344[1.153,1.566] | <0.001 | 1.356[1.158, 1.587] | 0.000 | 1.423[1.129, 1.553] | <0.001 |
| 2 | 1.409[1.168,1.696] | <0.001 | 1.552[1.278, 1.882] | 0.000 | 1.476[1.212, 1.794] | <0.001 |
| 3+ | 2.030[1.672,2.462] | <0.001 | 2.032[1.662, 2.483] | 0.000 | 1.926[1.571, 2.359] | <0.001 |
| *P* for trend | <0.001 |  | <0.001 |  | <0.001 |  |
| LCA driven ACE |  |  |  |  |  |  |
| Low risk | Ref |  | Ref ^a^ |  | Ref ^a1^ |  |
| Poor parent relationship | 2.207[1.726, 2.817] | <0.001 | 2.204[1.720, 2.820] | <0.001 | 2.108[1.637, 2.708] | <0.001 |
| Physical abuse | 1.380[1.169, 1.627] | <0.001 | 1.583[1.334, 1.877] | <0.001 | 1.483[1.246, 1.764] | <0.001 |
| Biological parent absence | 1.213[0.773, 1.873] | 0.391 | 1.112[0.700, 1.736] | 0.646 | 1.083[0.678, 1.703] | 0.733 |
| Mental neglect | 1.177[0.927, 1.489] | 0.178 | 1.180[0.924, 1.500] | 0.181 | 1.174[0.915, 1.499] | 0.204 |

Adjusted model 1: Adjusted for deprivation-related ACEs (if applicable), threat-related ACEs (if applicable), age, gender, marital status, education level,

Hukou, chronic disease, smoking status, drinking status, and house income. Adjusted model 2: Additionally adjusted for CES-D-10 scores at baseline based on model 1.

| Table S12 Associations between ACEs and the number of times with DSs according to gender in ordered-logistic regressions model. | | | | | | |
| --- | --- | --- | --- | --- | --- | --- |
|  | Crude model |  | Adjusted model 1 |  | Adjusted model 2 |  |
| Male |  |  |  |  |  |  |
| Total ACE | OR | *P* value | OR | *P* value | OR | *P* value |
| 0 | ref |  | ref |  | ref |  |
| 1 | 1.175[0.811, 1.721] | 0.399 | 1.111[0.729, 1.494] | 0.589 | 1.057[0.668, 1.446] | 0.780 |
| 2 | 1.020[0.705, 1.492] | 0.917 | 0.941[0.559, 1.323] | 0.755 | 0.869[0.481, 1.257] | 0.477 |
| 3+ | 1.862[1.327, 2.653] | <0.001 | 1.744[1.392, 2.096] | 0.002 | 1.575[1.217, 1.933] | 0.013 |
| *P* for trend | <0.001 |  | <0.001 |  | <0.001 |  |
| Deprivation-related ACE |  |  |  |  |  |  |
| 0 | ref |  | ref |  | ref |  |
| 1 | 1.324[0.992, 1.779] | 0.060 | 1.176[0.877, 1.476] | 0.288 | 1.097[0.793, 1.401] | 0.552 |
| 2 | 1.276[0.953, 1.722] | 0.106 | 1.096[0.792, 1.401] | 0.553 | 1.008[0.699, 1.317] | 0.960 |
| 3+ | 2.355[1.719, 3.246] | <0.001 | 1.878[1.548, 2.207] | 0.000 | 1.652[1.317, 1.987] | 0.003 |
| *P* for trend | <0.001 |  | 0.001 |  | 0.007 |  |
| Threat-related ACE |  |  |  |  |  |  |
| 0 | ref |  | ref |  | ref |  |
| 1 | 1.242[0.991, 1.554] | 0.058 | 1.206[0.975, 1.436] | 0.112 | 1.189[0.955, 1.423] | 0.147 |
| 2 | 1.459[1.134, 1.872] | 0.003 | 1.443[1.184, 1.702] | 0.005 | 1.409[1.146, 1.671] | 0.011 |
| 3+ | 1.935[1.489, 2.510] | <0.001 | 1.913[1.644, 2.182] | 0.000 | 1.859[1.587, 2.131] | 0.000 |
| *P* for trend | <0.001 |  | <0.001 |  | <0.001 |  |
| LCA driven ACE | |  |  |  |  |  |
| Low risk |  |  |  |  |  |  |
| Poor parent relationship | 2.131[1.490, 3.332] | <0.001 | 2.147[1.496, 3.062] | <0.001 | 2.183[1.513, 3.128] | <0.001 |
| Physical abuse | 1.561[1.260, 1.931] | <0.001 | 1.600[1.286, 1.987] | <0.001 | 1.510[1.210, 1.882] | <0.001 |
| Biological parent absence | 1.466[0.752, 2.747] | 0.244 | 1.306[0.662, 2.473] | 0.424 | 1.304[0.659, 2.482] | 0.429 |
| Mental neglect | 1.221[0.875, 1.719] | 0.261 | 1.188[0.830, 1.680] | 0.338 | 1.107[0.769, 1.577] | 0.577 |
| Female |  |  |  |  |  |  |
| Total ACE | OR | *P* value | OR | *P* value | OR | *P* value |
| 0 | ref |  | ref |  | ref |  |
| 1 | 1.318[0.963, 1.813] | 0.087 | 1.175[0.851, 1.499] | 0.330 | 1.124[0.796, 1.452] | 0.485 |
| 2 | 1.480[1.082, 2.036] | 0.015 | 1.307[0.983, 1.631] | 0.105 | 1.241[0.914, 2.452] | 0.196 |
| 3+ | 2.615[1.943, 3.542] | <0.001 | 2.373[2.067, 2.679] | 0.000 | 2.225[1.915, 2.534] | <0.001 |
| *P* for trend | <0.001 |  | <0.001 |  | <0.001 |  |
| Deprivation-related ACE |  |  |  |  |  |  |
| 0 | ref |  | ref |  | ref |  |
| 1 | 1.211[0.942, 1.560] | 0.137 | 1.099[0.838, 1.359] | 0.480 | 1.082[0.819, 1.346] | 0.557 |
| 2 | 1.508[1.167, 1.951] | 0.002 | 1.291[1.025, 1.557] | 0.060 | 1.300[1.032, 1.568] | 0.055 |
| 3+ | 2.520[1.894, 3.361] | <0.001 | 2.003[1.704, 2.303] | <0.001 | 1.939[1.637, 2.240] | <0.001 |
| *P* for trend | <0.001 |  | <0.001 |  | <0.001 |  |
| Threat-related ACE |  |  |  |  |  |  |
| 0 | ref |  | ref |  | ref |  |
| 1 | 1.513[1.222, 1.871] | <0.001 | 1.521[1.302, 1.740] | 0.000 | 1.478[1.256, 1.699] | 0.001 |
| 2 | 1.617[1.210, 2.155] | 0.001 | 1.699[1.400, 1.998] | 0.001 | 1.572[1.271, 1.873] | 0.003 |
| 3+ | 2.614[1.942, 3.517] | <0.001 | 2.144[1.838, 2.451] | 0.000 | 1.975[1.665, 2.285] | <0.001 |
| *P* for trend | <0.001 |  | <0.001 |  | <0.001 |  |
| LCA driven ACE |  |  |  |  |  |  |
| Low risk |  |  |  |  |  |  |
| Poor parent relationship | 2.348[1.665, 3.305] | <0.001 | 2.298[1.626, 3.241] | <0.001 | 2.101[1.48, 2.979] | <0.001 |
| Physical abuse | 1.547[1.174, 2.035] | 0.002 | 1.557[1.177, 2.055] | 0.002 | 1.459[1.099, 1.931] | 0.009 |
| Biological parent absence | 0.992[0.522, 1.829] | 0.981 | 1.000[0.520,1.868] | 0.999 | 0.965[0.497, 1.818] | 0.914 |
| Mental neglect | 1.149[0.824, 1.595] | 0.409 | 1.182[0.842, 1.650] | 0.330 | 1.247[0.883, 1.749] | 0.206 |

Adjusted model 1: Adjusted for deprivation-related ACEs (if applicable), threat-related ACEs (if applicable), age, marital status, education level, Hukou, chronic disease, smoking status, drinking status, and house income. Adjusted model 2: Additionally adjusted for CES-D-10 scores at baseline based on model 1.

| Table S13 Associations between ACEs and the number of times with DSs according to age in ordered-logistic regressions model. | | | | | | |
| --- | --- | --- | --- | --- | --- | --- |
|  | Crude model |  | Adjusted model 1 |  | Adjusted model 2 |  |
| 45-59 years old |  |  |  |  |  |  |
| Total ACE | OR | *P* value | OR | *P* value | OR | *P* value |
| 0 | ref |  | ref |  | ref |  |
| 1 | 1.233[0.934,1.636] | 0.142 | 1.179[0.891, 1.466] | 0.263 | 1.115[0.822, 1.409] | 0.466 |
| 2 | 1.192[0.903,1.581] | 0.218 | 1.123[0.835, 1.411] | 0.431 | 1.049[0.755, 1.343] | 0.750 |
| 3+ | 1.922[1.489,2.493] | <0.001 | 1.980[1.714, 2.246] | <0.001 | 1.747[1.475, 2.018] | <0.001 |
| P for trend | <0.001 |  | <0.001 |  | <0.001 |  |
| Deprivation-related ACE | |  |  |  |  |  |
| 0 | ref |  | ref |  | ref |  |
| 1 | 1.224[0.984,1.527] | 0.071 | 1.162[0.934, 1.390] | 0.198 | 1.085[0.853, 1.318] | 0.490 |
| 2 | 1.303[1.042,1.631] | 0.020 | 1.200[0.967, 1.433] | 0.125 | 1.144[0.907, 1.381] | 0.265 |
| 3+ | 2.439[1.901,3.136] | <0.001 | 2.019[1.757, 2.282] | 0.000 | 1.779[1.512, 2.046] | <0.001 |
| P for trend | <0.001 |  | <0.001 |  | <0.001 |  |
| Threat-related ACE |  |  |  |  |  |  |
| 0 | ref |  | ref |  | ref |  |
| 1 | 1.253[1.037,1.512] | 0.019 | 1.259[1.065, 1.454] | 0.020 | 1.214[1.017, 1.411] | 0.054 |
| 2 | 1.380[1.104,1.723] | 0.005 | 1.597[1.1365, 1.829] | <0.001 | 1.504[1.270, 1.739] | 0.001 |
| 3+ | 1.851[1.472,2.326] | <0.001 | 1.871[1.631, 2.111] | <0.001 | 1.751[1.508, 1.994] | <0.001 |
| *P* for trend | <0.001 |  | <0.001 |  | <0.001 |  |
| LCA driven ACE |  |  |  |  |  |  |
| Low risk | ref |  | ref |  | ref |  |
| Poor parent relationship | 2.003[1.495, 2.678] | <0.001 | 2.106[1.565, 2.824] | <0.001 | 1.968[1.458, 2.648] | <0.001 |
| Physical abuse | 1.356[1.116, 1.647] | 0.002 | 1.583[1.293,1.936] | 0.000 | 1.467[1.194, 1.800] | <0.001 |
| Biological parent absence | 0.777[0.408, 1.410] | 0.422 | 0.716[0.369, 1.326] | 0.303 | 0.715[0.346, 1.340] | 0.310 |
| Mental neglect | 1.154[0.853, 1.551] | 0.348 | 1.174[0.862, 1.589] | 0.302 | 1.160[0.848, 1.576] | 0.348 |
| ≥60 years old |  |  |  |  |  |  |
| Total ACE | OR | *P* value | OR | *P* value | OR | *P* value |
| 0 | ref |  | ref |  | ref |  |
| 1 | 1.323[0.824,2.175] | 0.256 | 1.247[0.765, 2.077] | 0.385 | 1.153[0.704, 1.927] | 0.580 |
| 2 | 1.362[0.851,2.230] | 0.208 | 1.305[0.803, 2.168] | 0.292 | 1.215[0.744, 2.025] | 0.445 |
| 3+ | 2.461[1.571,3.959] | <0.001 | 2.524[1.587, 4.118] | 0.000 | 2.348[1.471, 3.843] | <0.001 |
| *P* for trend | <0.001 |  | <0.001 |  | <0.001 |  |
| Deprivation-related ACE | |  |  |  |  |  |
| 0 | ref |  | ref |  | ref |  |
| 1 | 1.175[0.811, 1.720] | 0.400 | 1.169[0.794, 1.741] | 0.434 | 1.168[0.790, 1.746] | 0.441 |
| 2 | 1.320[0.909, 1.937] | 0.150 | 1.258[0.850, 1.879] | 0.256 | 1.249[0.841, 1.874] | 0.275 |
| 3+ | 2.122[1.428, 3.182] | <0.001 | 1.872[1.235, 2.863] | 0.003 | 1.845[1.212, 2.833] | 0.005 |
| *P* for trend | <0.001 |  | 0.001 |  | 0.002 |  |
| Threat-related ACE |  |  |  |  |  |  |
| 0 | ref |  |  |  |  |  |
| 1 | 1.531[1.176,1.989] | 0.001 | 1.510[1.147, 1.984] | 0.003 | 1.521[1.153, 2.002] | 0.003 |
| 2 | 1.427[1.010,2.004] | 0.042 | 1.400[0.968, 2.010] | 0.071 | 1.369[0.944, 1.973] | 0.094 |
| 3+ | 2.479[1.718,3.570] | <0.001 | 2.553[1.745, 3.723] | <0.001 | 2.510[1.712, 3.669] | <0.001 |
| *P* for trend | <0.001 |  | <0.001 |  | <0.001 |  |
| LCA driven ACE |  |  |  |  |  |  |
| Low risk | ref |  | ref |  | ref |  |
| Poor parent relationship | 2.733[1.734, 4.290] | <0.001 | 2.545[1.607, 4.009] | <0.001 | 2.559[1.595, 4.080] | <0.001 |
| Physical abuse | 1.388[1.009, 1.898] | 0.042 | 1.656[1.193, 2.288] | 0.002 | 1.585[1.139, 2.194] | 0.006 |
| Biological parent absence | 2.167[1.118, 4.125] | 0.020 | 1.797[0.918, 3.452] | 0.081 | 1.774[0.895, 3.450] | 0.094 |
| Mental neglect | 1.231[0.827, 1.811] | 0.297 | 1.199[0.799, 1.781] | 0.373 | 1.217[0.806, 1.815] | 0.342 |

Adjusted model 1: Adjusted for deprivation-related ACEs (if applicable), threat-related ACEs (if applicable), gender, marital status, education level,

Hukou, chronic disease, smoking status, drinking status, and house income. Adjusted model 2: Additionally adjusted for CES-D-10 scores at baseline based on model 1.
